# Myosin VI-Dependent Actin Cages Encapsulate Parkin-Positive Damaged Mitochondria

**DOI:** 10.1016/j.devcel.2018.01.007

**Published:** 2018-02-26

**Authors:** Antonina J. Kruppa, Chieko Kishi-Itakura, Thomas A. Masters, Joanna E. Rorbach, Guinevere L. Grice, John Kendrick-Jones, James A. Nathan, Michal Minczuk, Folma Buss

**Affiliations:** 1 Cambridge Institute for Medical Research, Department of Clinical Biochemistry, University of Cambridge, Cambridge Biomedical Campus, Wellcome Trust/MRC Building, Hills Road, Cambridge, CB2 0XY, UK; 2 MRC Mitochondrial Biology Unit, University of Cambridge, Cambridge Biomedical Campus, Wellcome Trust/MRC Building, Hills Road, Cambridge, CB2 0XY, UK; 3 Cambridge Institute for Medical Research, Department of Medicine, University of Cambridge, Cambridge Biomedical Campus, Wellcome Trust/MRC Building, Hills Road, Cambridge, CB2 0XY, UK; 4 MRC Laboratory of Molecular Biology, Francis Crick Avenue, Cambridge Biomedical Campus, Cambridge, CB2 0QH, UK

**Keywords:** MYO6, myosin VI, actin, mitophagy, Parkin, mitochondrial quality control, TAX1BP1, NDP52, OPTN

## Abstract

Mitochondrial quality control is essential to maintain cellular homeostasis and is achieved by removing damaged, ubiquitinated mitochondria via Parkin-mediated mitophagy. Here, we demonstrate that MYO6 (myosin VI), a unique myosin that moves toward the minus end of actin filaments, forms a complex with Parkin and is selectively recruited to damaged mitochondria via its ubiquitin-binding domain. This myosin motor initiates the assembly of F-actin cages to encapsulate damaged mitochondria by forming a physical barrier that prevents refusion with neighboring populations. Loss of MYO6 results in an accumulation of mitophagosomes and an increase in mitochondrial mass. In addition, we observe downstream mitochondrial dysfunction manifesting as reduced respiratory capacity and decreased ability to rely on oxidative phosphorylation for energy production. Our work uncovers a crucial step in mitochondrial quality control: the formation of MYO6-dependent actin cages that ensure isolation of damaged mitochondria from the network.

## Highlights

• MYO6 forms a complex with Parkin and is recruited to damaged mitochondria
• MYO6 triggers F-actin cage assembly around dysfunctional mitochondria
• Actin cages form a barrier thereby preventing refusion of damaged mitochondria
• Loss of MYO6 causes an accumulation of mitophagosomes and mitochondrial dysfunction

## Graphical Abstract

**Figure:**
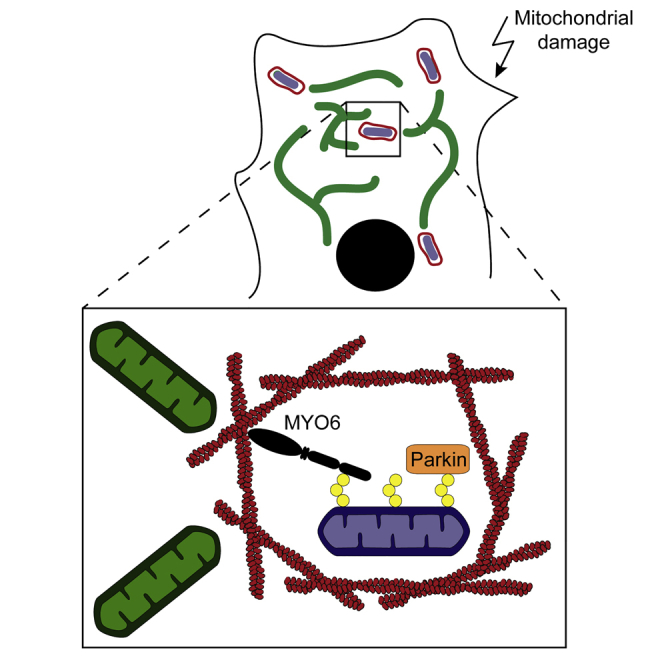

## Teaser

Kruppa et al. demonstrate that MYO6 forms a complex with Parkin and is recruited to damaged, ubiquitinated mitochondria. MYO6 promotes F-actin cage assembly to isolate and prevent refusion of dysfunctional organelles. Loss of MYO6 leads to a mitophagy defect with an accumulation of mitophagosomes and downstream mitochondrial dysfunction.

## Introduction

Mitochondrial homeostasis involves the constant remodeling of the mitochondrial network through fission and fusion events, but also requires the isolation and subsequent removal of dysfunctional mitochondria from this dynamic network. Damaged organelles are then targeted for clearance via a specialized selective autophagy pathway termed mitophagy (Narendra et al., 2008). The E3 ubiquitin ligase Parkin (PARK2) is a key regulator of the mitophagy pathway, and several forms of autosomal recessive Parkinson's disease are caused by mutations in Parkin (Ryan et al., 2015). A hallmark of mitochondrial damage is the loss of membrane potential resulting in recruitment of Parkin to the outer mitochondrial membrane (OMM), which extensively ubiquitinates OMM proteins thereby amplifying the mitophagy signal (Nguyen et al., 2016). Cargo-specific autophagy receptors (OPTN, NDP52, and TAX1BP1) then recognize and capture ubiquitinated mitochondria via their ubiquitin-binding domains and simultaneously bind to LC3 on autophagosomal membranes via their LC3 interacting region motif (Lazarou et al., 2015, Moore and Holzbaur, 2016, Rogov et al., 2014, Wong and Holzbaur, 2014). Finally, the dysfunctional, ubiquitinated mitochondria are sequestered into double-membraned structures called autophagosomes, forming mitophagosomes, which ultimately fuse with lysosomes for degradation (Ktistakis and Tooze, 2016).

We have previously shown that TAX1BP1, NDP52, and OPTN directly bind to myosin VI (MYO6) (Morriswood et al., 2007, Sahlender et al., 2005), a unique highly specialized motor protein that moves toward the minus end of actin filaments, in the opposite direction to all other myosins (Wells et al., 1999). This motor plays essential roles in regulating actin organization (Mangold et al., 2011, Noguchi et al., 2006), and functions in intracellular cargo sorting and vesicle transport linked to endocytosis and autophagy (Buss et al., 1998, Buss et al., 2001, Dance et al., 2004, Spudich et al., 2007, Tumbarello et al., 2012, Warner et al., 2003). Specifically, we have shown that MYO6 is important in non-selective and selective autophagy pathways for the clearance of invading pathogens (xenophagy) and protein aggregates (aggrephagy) (Tumbarello et al., 2012, Tumbarello et al., 2015). These autophagy defects were also observed in cells derived from the *Snell's waltzer* mouse, which lacks MYO6 due to a spontaneous intragenic deletion (Avraham et al., 1995, Tumbarello et al., 2012).

The intracellular localization and functions of MYO6 are mediated by cargo adaptor proteins, which bind to specific sites in the C-terminal cargo-binding domain (CBD) of the tail via either an RRL motif (NDP52, OPTN, TAX1BP1, and GIPC) or a WWY motif (TOM1, LMTK2, and DAB2) (Bunn et al., 1999, Chibalina et al., 2007, Morris et al., 2002, Morriswood et al., 2007, Sahlender et al., 2005, Spudich et al., 2007, Tumbarello et al., 2012). In addition, the tail of MYO6 can bind to ubiquitin and contains a phospholipid-binding domain (He et al., 2016, Penengo et al., 2006, Spudich et al., 2007). Using an unbiased mass spectrometry approach, MYO6 and its endocytic cargo adaptor, TOM1, were identified as proteins that associate with Parkin in response to mitochondrial damage (Sarraf et al., 2013).

Taken together, this suggests a crucial link between MYO6 and its adaptor proteins to mitochondrial quality control mechanisms including Parkin-mediated mitophagy. In this study, we demonstrate that MYO6 is recruited via its ubiquitin-binding domain and independently from the autophagy receptors to damaged mitochondria by a Parkin-dependent mechanism. We define a new quality-control step during mitophagy in which MYO6, together with the actin regulator, cdc42, and actin nucleators (Arp2/3 complex, formins, and N-WASP), promotes the assembly of F-actin cages to encapsulate damaged mitochondria within hours of the mitochondrial insult inhibiting their refusion with neighboring populations. In addition, MYO6 functions in the final stages of the pathway mediating the clearance of damaged mitochondria via autophagy, as loss of MYO6 leads to an accumulation of autophagosomes containing mitochondria. We observe that the absence of MYO6 leads to profound mitochondrial dysfunction, as cells lacking MYO6 accumulate defective mitochondria. Hence, our evidence suggests that MYO6 is a novel player in mitochondrial quality control and maintenance of mitochondrial homeostasis.

## Results

### MYO6 Is Recruited to Damaged Mitochondria and Interacts with Parkin

**Figure 1:**
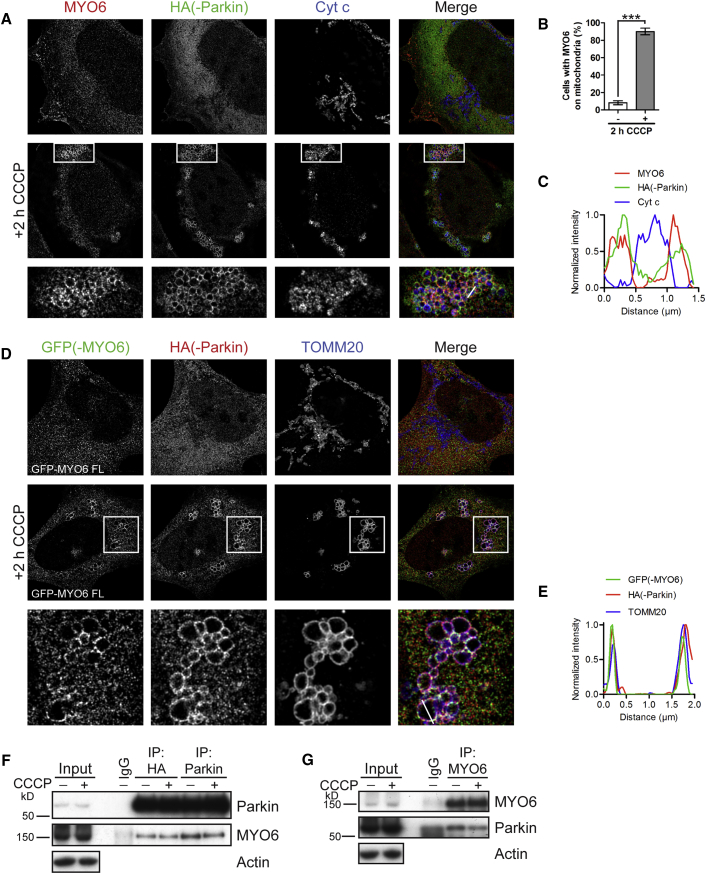
Endogenous and GFP-Tagged MYO6 Are Recruited to Damaged Mitochondria and Form a Complex with Parkin (A) HA-Parkin-expressing HEK293 cells were treated for 2 h with 10 μM CCCP or left untreated. Images were acquired by superresolution structured illumination microscopy (SR-SIM) after staining for endogenous MYO6, HA to detect Parkin, and cytochrome c (Cyt c) to visualize mitochondria. (B) Quantitation of the percentage of cells with endogenous MYO6 on Cyt c-labeled mitochondria from (A) by widefield microscopy. Data are represented as mean ± SEM. Two-tailed unpaired Student's t test, ^∗∗∗^p < 0.001, n = 3 (≥427 cells per condition). (C) Line profile of MYO6- and Parkin-positive mitochondrion along the white line indicated in (A). (D) HEK293 cells stably expressing HA-Parkin transiently transfected with full-length (FL) GFP-MYO6 were left untreated or incubated for 2 h with 10 μM CCCP. Images were acquired by SR-SIM after staining for the GFP tag on MYO6, HA to detect Parkin, and TOMM20 to label the outer mitochondrial membrane. (E) Line profile of MYO6- and Parkin-positive mitochondrion along the white line indicated in (D). (F) Parkin was immunoprecipitated using antibodies either against the HA tag or Parkin from HA-Parkin-expressing HEK293 cells incubated for 1 h with 10 μM CCCP or left untreated. The inputs, control immunoglobulin G (IgG) immunoprecipitation (IP), and HA/Parkin IPs were immunoblotted for Parkin as well as co-immunoprecipitation of endogenous MYO6. Actin is shown as a loading control. (G) Endogenous MYO6 was immunoprecipitated from HA-Parkin-expressing HEK293 cells incubated for 1 h with 10 μM CCCP or left untreated. The inputs, IgG control IP, and MYO6 IPs were immunoblotted for MYO6 as well as co-immunoprecipitation of Parkin. Actin is shown as a loading control. Images in (A), (D), (F), and (G) are representative of three independent experiments. See also Figure S1.

First, we investigated whether MYO6 plays a role in the clearance of damaged mitochondria by Parkin-mediated mitophagy. Mitochondrial damage was induced either by treating cells with the protonophore, carbonyl cyanide 3-chlorophenylhydrazone (CCCP), causing depolarization or by using the electron transport chain complex III inhibitor, antimycin A, in combination with oligomycin (an ATP synthase inhibitor), which prevents mitochondrial repolarization. Both treatments cause fragmentation of the mitochondrial network and Parkin relocalization from the cytoplasm to the OMM (Narendra et al., 2008). Using superresolution structured illumination microscopy (SR-SIM), we observed that endogenous MYO6, which normally resides on intracellular vesicles, the plasma membrane, and in the cytosol (Buss et al., 1998, Chibalina et al., 2007, Tumbarello et al., 2012, Warner et al., 2003), was strongly recruited to and colocalized with Parkin-positive damaged mitochondria stained for cytochrome c after 2 h of CCCP treatment in ∼90% of HEK293 cells expressing Parkin (Figures 1A–1C) or after 3 h treatment with oligomycin/antimycin A (OA) (Figure S1A).

GFP-tagged MYO6 also relocalized to the Parkin-positive ring-like OMM and strongly colocalized with TOMM20 after CCCP (Figures 1D and 1E) and also after OA treatment (Figure S1B). MYO6 recruitment was Parkin dependent, since this motor was not recruited to damaged mitochondria in HEK293 cells expressing Parkin, with the C431S mutation resulting in a catalytically inactive E3 ligase that no longer targets to mitochondria (Figure S1C).

To further analyze the link between MYO6 and Parkin, we performed co-immunoprecipitation studies with antibodies to Parkin or the hemagglutinin (HA) tag. Our results demonstrate that Parkin forms a complex with MYO6 independent of CCCP treatment (Figure 1F), which we also observed when we performed the reverse by immunoprecipitating endogenous MYO6 (Figure 1G). Interestingly, the Parkin-MYO6 complex did not involve the mitophagy receptors, OPTN, NDP52, and TAX1BP1, as these were not co-immunoprecipitated with Parkin (data not shown).

Taken together, we show that endogenous as well as ectopically expressed MYO6 translocate to the OMM upon mitochondrial stress in a Parkin-dependent manner and also associate with Parkin independently of mitophagy induction and the mitophagy receptors.

### The Ubiquitin- and Autophagy Receptor-Binding Domain of MYO6 Is Required for Parkin-Dependent Recruitment to Damaged Mitochondria

**Figure 2:**
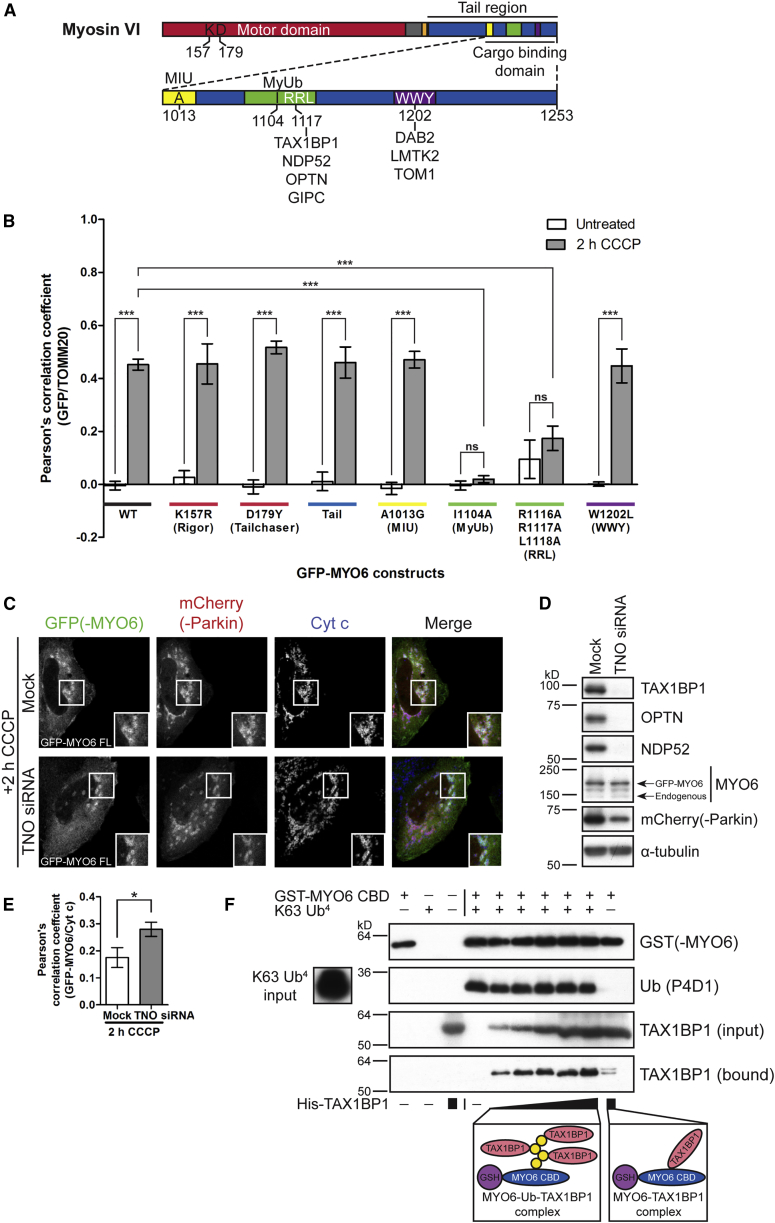
MYO6 Binding to Ubiquitin, but Not to the Autophagy Receptors, Is Required for Parkin-Dependent Recruitment to Damaged Mitochondria (A) Illustration of MYO6 domain organization: catalytic motor domain (red), unique insert “reverse gear” (gray), IQ calmodulin-binding motif (orange), and a cargo-binding domain (CBD) in the tail region (blue). Enlarged is the CBD containing two protein-protein interaction motifs (RRL, green; WWY, purple) and ubiquitin-binding domains (motif interacting with ubiquitin [MIU], yellow; MYO6 ubiquitin-binding domain [MyUb], green). Amino acid residues of single point mutations in different domains are highlighted. (B) Quantitation of the degree of colocalization between the GFP-MYO6 constructs (wild-type [WT] or the indicated mutants) and TOMM20-labeled mitochondria in HeLaM cells stably expressing HA-Parkin left untreated or incubated for 2 h with 10 μM CCCP by determining the Pearson's correlation coefficient of confocal microscopy images. Data are represented as mean ± SEM. One-way ANOVA with *post-hoc* Bonferroni correction, ^∗∗∗^p < 0.001, n ≥ 3 (≥50 cells per condition). (C) HeLaM cells were treated with siRNA against TAX1BP1, NDP52, and OPTN (TNO), then transiently transfected with GFP-MYO6 FL as well as mCherry-Parkin and incubated for 2 h with 10 μM CCCP. Images were acquired by confocal microscopy after staining the GFP tag on MYO6, the mCherry tag on Parkin with a DsRed antibody, and Cyt c to visualize mitochondria. (D) Western blot analysis of lysates corresponding to (C) confirming depletion of TAX1BP1, NDP52, and OPTN, as well as overexpression of GFP-MYO6 and mCherry-Parkin. α-Tubulin is shown as a loading control. Images in (C) and (D) are representative of three independent experiments. (E) Quantitation of the degree of colocalization between GFP-MYO6 and Cyt c-labeled mitochondria by determining the Pearson's correlation coefficient of confocal microscopy images from (C). Data are represented as mean ± SEM. Two-tailed paired Student's t test, ^∗^p < 0.05, n = 3 (≥100 cells per condition). (F) Competition assay where GST-tagged MYO6 CBD (blue) and K63 tetra-ubiquitin (Ub^4^) chains (yellow) were pre-incubated with glutathione (GSH) sepharose (purple) and then incubated with increasing amounts of His-tagged TAX1BP1 (C-terminal half, pink). The samples were analyzed by immunoblotting with antibodies against GST to detect MYO6, bound ubiquitin (P4D1), and bound TAX1BP1. As controls, GST-MYO6 CBD, K63 Ub^4^, and His-TAX1BP1 were individually incubated with GSH sepharose to determine if there was any non-specific binding. GST-MYO6 CBD and His-TAX1BP1 were also incubated together to demonstrate direct binding. Inputs of K63 Ub^4^ and His-TAX1BP1 (TAX1BP1 input) are also shown. Images are representative of four independent experiments. See also Figures S2–S4.

To delineate which functional domain or protein-protein interaction motif in MYO6 is responsible for recruitment to damaged mitochondria, we transiently expressed GFP-tagged MYO6 constructs with specific point mutations in HeLaM cells and assessed their level of recruitment to mitochondria by quantifying the degree of colocalization between the GFP and TOMM20 channels (Figures 2A and 2B).

We first analyzed mutations in the motor domain: the K157R mutation (rigor) in the ATP-binding site (P loop) of MYO6 that results in increased actin binding (Aschenbrenner et al., 2004) and the D179Y mutation (tailchaser) in the transducer, which leads to premature inorganic phosphate release from the head (Pylypenko et al., 2015). Both motor domain mutants and the tail of MYO6 were sufficient for recruitment of MYO6 to mitochondria, implying that motor activity is not required for this process (Figures 2B and S2). Point mutants in the motif interacting with ubiquitin (A1013G) (Penengo et al., 2006) or the WWY motif (W1202L) important for binding to DAB2, LMTK2, and TOM1 (Chibalina et al., 2007, Morris et al., 2002, Spudich et al., 2007, Tumbarello et al., 2012), located in the CBD of the MYO6 tail region are still recruited to damaged mitochondria. In contrast, mutating the RRL motif (R1116A/R1117A/L1118A) responsible for interactions with the autophagy receptors (TAX1BP1, OPTN, and NDP52) and GIPC (Bunn et al., 1999, Morriswood et al., 2007, Sahlender et al., 2005) or the MYO6 ubiquitin-binding domain (MyUb, I1104A) (He et al., 2016) completely abolished recruitment of GFP-tagged mutant MYO6 to damaged mitochondria (Figures 2B and S2).

In brief, these results demonstrate that MYO6 recruitment does not require motor activity, but rather depends on a protein-protein interaction region in the tail domain that mediates binding to autophagy receptors and/or ubiquitin.

### MYO6 and the Autophagy Receptors Are Independently Recruited to Damaged Mitochondria via Ubiquitin

We next elucidated whether binding of MYO6 to its known adaptor proteins (the autophagy receptors), ubiquitin, or both were responsible for recruiting MYO6 to damaged mitochondria.

The MYO6 binding partners TAX1BP1, OPTN, and NDP52 have previously been shown to be recruited to damaged mitochondria as overexpressed and tagged proteins (Heo et al., 2015, Lazarou et al., 2015, Moore and Holzbaur, 2016, Wong and Holzbaur, 2014). We followed the relocalization of the endogenous proteins and found that TAX1BP1, similar to MYO6, is recruited to the majority of Parkin-positive mitochondria, whereas OPTN, and particularly NDP52, are recruited to foci on mitochondria (Figure S3). Recruitment of the mitophagy receptors does not require MYO6, since endogenous TAX1BP1, NDP52, and also OPTN, are still targeted to damaged mitochondria in CRISPR/Cas9 MYO6 knockout (KO) HeLaM cells (Figures S4A–S4D).

We subsequently analyzed whether binding of MYO6 to the mitophagy receptors was required for bringing MYO6 to damaged mitochondria by performing a triple small interfering RNA (siRNA) knock down of TAX1BP1, NDP52, and OPTN (TNO) in HeLaM cells. The efficient depletion of all three mitophagy receptors enhanced GFP-MYO6 recruitment to damaged mitochondria (Figures 2C–2E), possibly indicating that, in the absence of the mitophagy receptors, more ubiquitin chains attached to OMM proteins may be freely available to MYO6. We have previously demonstrated that the ubiquitin-binding domain overlaps with the MYO6-interacting region in all three mitophagy receptors (see Figure S4E for TAX1BP1 schematic), and that single-point mutations that inhibit ubiquitin binding also ablate MYO6 binding (Tumbarello et al., 2015). Therefore, while bound to ubiquitin on the mitochondrial surface, TAX1BP1, NDP52, or OPTN are no longer able to recruit MYO6 to mitochondria, and MYO6 instead relies on its ubiquitin-binding (MyUb) domain. The observed loss in recruitment of MYO6 to damaged mitochondria by mutating the RRL motif (Figure 2B) is likely to be linked to destabilization of the MyUb domain, as R^1117^ has been shown to be important for structural integrity (He et al., 2016).

Having established that MYO6 is recruited to damaged mitochondria via its ubiquitin-binding domain, we next performed a detailed analysis of MYO6 binding to polyubiquitin chains (Figure S4). We assessed the ability of purified His-tagged CBD of MYO6 to bind ubiquitin chains of defined length *in vitro*. MYO6 bound strongly to K63 tetra-ubiquitin (Ub^4^) chains, but little binding to K11 Ub^4^ chains was observed (Figure S4F), in agreement with published data using di-ubiquitin linkages (He et al., 2016). Similarly, His-tagged TAX1BP1 also has a stronger preference for binding to K63 compared with K11 Ub^4^ chains (Figure S4G). Hence, MYO6 and TAX1BP1 both have the ability to bind K63 and, to a lesser extent, K11 ubiquitin chains *in vitro*. Since the mitophagy receptor and ubiquitin-binding sites overlap and are likely to be exclusive in the MYO6 CBD (He et al., 2016, Morriswood et al., 2007, Sahlender et al., 2005), we tested whether increasing concentrations of His-TAX1BP1 are able to compete with GST-MYO6 CBD prebound to K63 Ub^4^ chains *in vitro*. Interestingly, we found that increasing amounts of TAX1BP1 did not displace MYO6 CBD from the ubiquitin chains, but instead bound to the K63 Ub^4^ chains forming a MYO6-ubiquitin-TAX1BP1 complex (Figure 2F).

In summary, MYO6 and the mitophagy receptors, such as TAX1BP1, are recruited independently and in parallel to ubiquitin on the surface of damaged mitochondria, since their binding sites are mutually exclusive. Thus, MYO6 appears to play a role during mitophagy that is independent from, and does not require, associations with the autophagy receptors.

### MYO6 and Actin Regulators/Nucleators Assemble F-Actin Cages around Damaged Mitochondria

**Figure 3:**
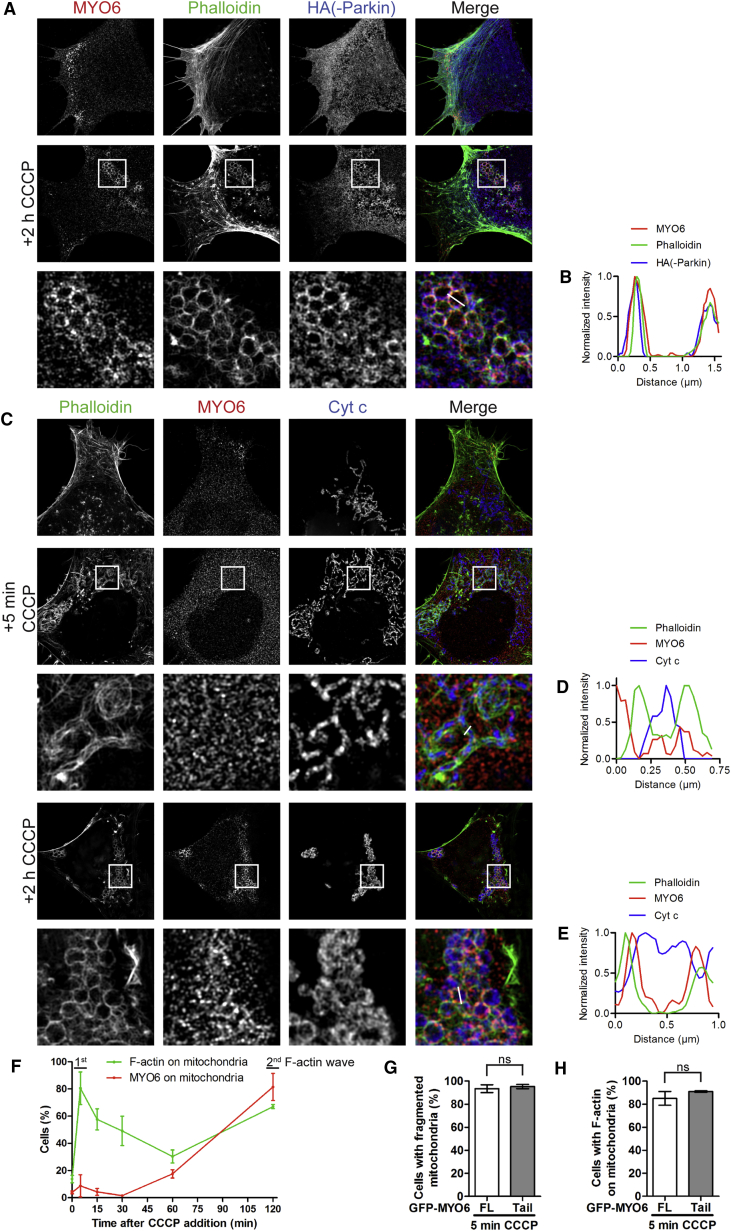
Time Course of MYO6 and F-Actin Assembly around Damaged Mitochondria (A) HEK293 cells stably expressing HA-Parkin were left untreated or incubated for 2 h with 10 μM CCCP. Images were acquired by SR-SIM after staining for endogenous MYO6, F-actin was visualized with phalloidin, and HA to detect Parkin. (B) Line profile of MYO6- and Parkin-positive mitochondrion that is F-actin-positive along the white line indicated in (A). (C) HA-Parkin-expressing HEK293 cells were left untreated or incubated for 5 min and 2 h with 10 μM CCCP. Images were acquired by SR-SIM after staining for endogenous MYO6, F-actin was visualized with phalloidin, and Cyt c as a mitochondrial marker. (D and E) Line profiles of F-actin-positive mitochondria along the white lines indicated in (C) that are MYO6-negative after 5 min CCCP treatment (D) or MYO6-positive after 2 h CCCP incubation (E). (F) Quantitation of the percentage of cells with endogenous MYO6 or F-actin on Cyt c-labeled mitochondria from (C) at the indicated time points by widefield microscopy. Data are represented as mean ± SEM, n = 3 (≥299 cells per condition). (G and H) Quantitation of the percentage of HA-Parkin-expressing HeLaM cells transiently transfected with GFP-MYO6, either FL or tail, incubated for 5 min with 10 μM CCCP (Figure S5C) that have F-actin on mitochondria (G) and fragmented Cyt c-labeled mitochondria (H) by widefield microscopy. Data are represented as mean ± SEM. Two-tailed paired Student's t test, ns, not significant, n = 3 (≥213 cells per condition). Images in (A) and (C) are representative of three independent experiments. See also Figure S5.

Myosin motor proteins not only translocate along actin filaments to generate movement and force, but they also actively regulate the dynamic turnover of the actin cytoskeleton. We therefore investigated whether MYO6 recruitment was accompanied by the assembly of an F-actin network around mitochondria in cells stained with phalloidin. Using SR-SIM, we observed a dramatic reorganization of the actin network and assembly of intricate F-actin cages on Parkin-positive structures that surround damaged mitochondria in HEK293 cells expressing Parkin treated for 2 h with CCCP (Figures 3A, 3B, S5A, and S5B). The assembly of these actin cages around damaged mitochondria was also observed in HEK293 cells with endogenous Parkin after 6 h of CCCP treatment (Figures 5A and 5B).

The actin cytoskeleton has been reported to play an important role in mitochondrial fission. Transient actin polymerization on the OMM is known to occur within 2–5 min after C/FCCP treatment, and these short-lived (<15 min) actin filaments are required for mitochondrial fission (Korobova et al., 2013, Li et al., 2015). To determine the kinetics of MYO6 and F-actin recruitment to damaged mitochondria, we performed a detailed time course experiment to visualize MYO6 and F-actin assembly on damaged mitochondria over time. Five minutes after CCCP addition, we observed a rapid assembly of F-actin on mitochondria in ∼80% of cells, but no recruitment of endogenous as well as GFP-tagged full-length MYO6 or the dominant-negative tail (Figures 3C, 3D, 3F, and S5C). F-actin levels then drop and only after prolonged treatment with CCCP (>1 h) is there a strong increase in cells with MYO6 present on mitochondria concomitant with a rise in F-actin by 2 h (Figures 3C–3F). These data demonstrate that there are two waves of F-actin assembly on mitochondria, the first one after 5 min independent of, and the second one after 2 h dependent on, MYO6. This result is supported by the finding that overexpression of the dominant-negative MYO6 tail does not inhibit the assembly of mitochondrial F-actin induced after 5 min of CCCP treatment or the process of mitochondrial fission; indeed, the mitochondrial morphology of MYO6 tail-expressing cells under steady-state conditions is network-like and similar to cells expressing GFP-MYO6 FL (Figures 3G, 3H, and S5C). In short, although actin filaments are essential for mitochondrial fission, we demonstrate that MYO6 is not involved in this process. Mitochondrial recruitment of MYO6 correlates only with the second wave of actin assembly at a much later time point, which is consistent with this being a distinct pathway.

**Figure 4:**
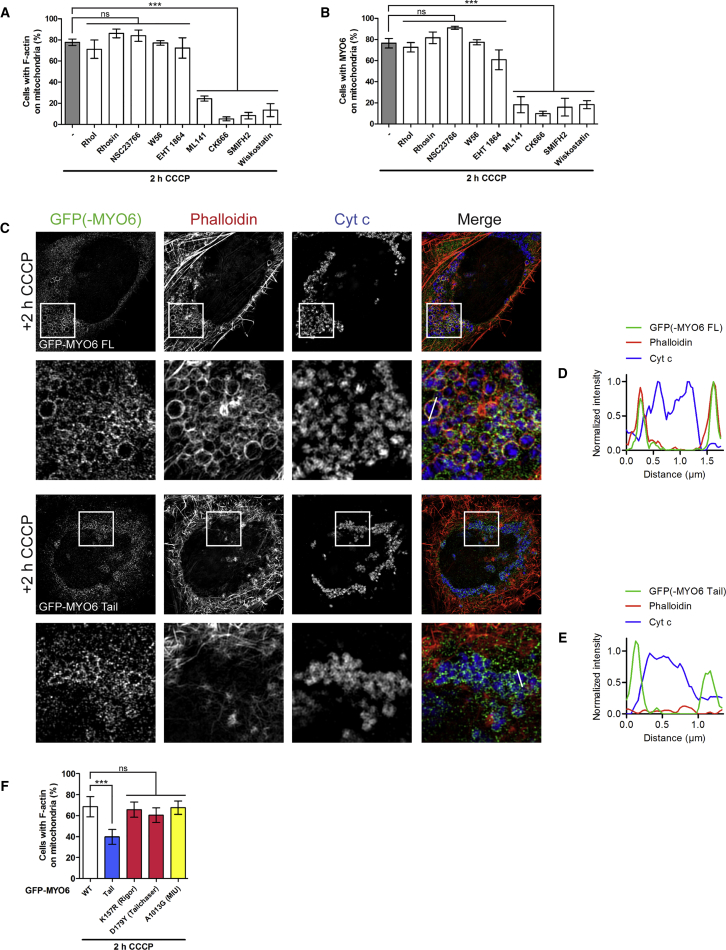
MYO6 and Actin Regulators/Nucleators Mediate the Assembly of F-Actin Cages around Damaged Mitochondria (A and B) HEK293 cells stably expressing HA-Parkin were incubated for 2 h with 10 μM CCCP and inhibitors of actin regulators Rho (0.5 μg/mL Rho inhibitor I, RhoI, or 50 μM Rhosin), Rac1 (100 μM NSC23766, 100 μM W56, or 10 μM EHT 1864), or cdc42 (20 μM ML141), and actin nucleators Arp2/3 complex (100 μM CK666), formins (20 μM SMIFH2), or N-WASP (5 μM Wiskostatin) (Figure S6). Quantitation of the percentage of cells with F-actin (A) and endogenous MYO6 (B) on mitochondria by widefield microscopy. Data are represented as mean ± SEM. One-way ANOVA with *post-hoc* Bonferroni correction, ^∗∗∗^p < 0.001, ns, not significant, n ≥ 3 (≥339 cells per condition). (C) HeLaM cells stably expressing HA-Parkin transiently transfected with GFP-MYO6, either FL or tail, were incubated for 2 h with 10 μM CCCP. Images were acquired by SR-SIM after staining for the GFP tag on MYO6, with phalloidin to visualize F-actin, and Cyt c as a mitochondrial marker. Images are representative of three independent experiments. (D and E) Line profiles of GFP-MYO6-positive mitochondria along the white lines indicated in (C) that are actin-positive in the case of GFP-MYO6 FL (D) or actin-negative for the tail (E). (F) Quantitation of the percentage of HA-Parkin-expressing HeLaM cells expressing GFP-MYO6, either FL WT, tail, or the indicated mutants, incubated for 2 h with 10 μM CCCP that have F-actin on mitochondria by widefield microscopy. Data are represented as mean ± SEM. One-way repeated measures ANOVA with *post-hoc* Bonferroni correction, ^∗∗∗^p < 0.001, ns, not significant, n = 3 (≥241 cells per condition). See also Figure S6.

To determine which actin regulators and nucleators are involved in the assembly of F-actin cages around damaged mitochondria, we used a panel of inhibitors against actin regulators (Rho [RhoI or Rhosin], Rac1 [NSC23766, W56 or EHT 1864], and cdc42 [ML141]) and nucleators including the Arp2/3 complex (CK666), formins (SMIFH2), and N-WASP (Wiskostatin). Loss of cdc42 activity significantly reduced actin cage formation as well as MYO6 recruitment around damaged mitochondria after 2 h CCCP treatment, whereas inhibition of Rho and Rac1 using several different inhibitors had no effect (Figures 4A, 4B, and S6). Moreover, the Arp2/3 complex, formins, and N-WASP appear to be critical nucleators for actin cage assembly around damaged mitochondria as their inhibition led to a complete loss of actin and MYO6 on mitochondria in the majority of cells (Figures 4A, 4B, and S6).

Furthermore, actin filament assembly around Parkin-positive mitochondria after 2 h CCCP treatment requires full-length MYO6 (Figures 4C and 4D), since overexpression of the dominant-negative MYO6 tail, which still targets to mitochondria (Figures 2B and S2) but can no longer bind to actin, inhibits the formation of F-actin cages around damaged mitochondria (Figures 4C, 4E, and 4F). Unsurprisingly, MYO6 mutations, such as K157R, D179Y, or A1013G, which target to mitochondria and still bind to actin, do not inhibit F-actin assembly on damaged mitochondria (Figure 4F).

Taken together, these data show that MYO6 plays a crucial role in the assembly of F-actin cages around damaged mitochondria, which is regulated by the Rho GTPase, cdc42, as well as downstream nucleators, such as the Arp2/3 complex, formins, and N-WASP. Our results demonstrate that two distinct pathways for F-actin assembly on mitochondria after CCCP treatment exist: a first wave (within minutes) of transient mitochondrial F-actin structures that mediate mitochondrial fission, which is MYO6 independent, followed by a second wave (after 2 h) leading to the formation of stable MYO6-dependent F-actin cages around damaged mitochondria after mitophagy induction concurrent with the recruitment of Parkin.

### F-Actin Cages Inhibit Refusion of Fragmented Mitochondria

**Figure 5:**
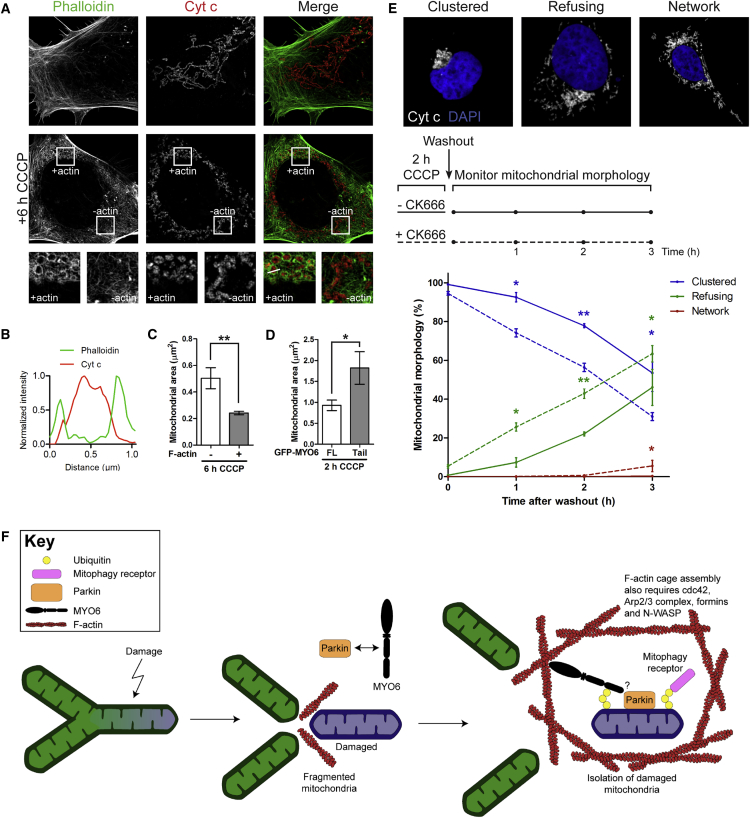
F-Actin Cages around Mitochondria Restrict Fragment Size and Lead to a Reduced Refusion Rate (A) HEK293 cells with endogenous Parkin were left untreated or incubated for 6 h with 10 μM CCCP. Images were acquired by SR-SIM after staining F-actin with phalloidin and Cyt c to label mitochondria. Images are representative of three independent experiments. (B) Line profile of F-actin-positive mitochondrion along the white line in (A). (C) Quantitation of the area of mitochondria that are actin positive (+) or negative (–) in regions of interest from cells imaged in (A). Data are represented as mean ± SEM. Two-tailed paired Student's t test, ^∗∗^p < 0.01, n = 17 from three independent experiments. (D) Quantitation of the area of mitochondria in regions of interest with GFP-MYO6 FL (with actin) or tail (without actin) recruitment in HA-Parkin-expressing HeLaM cells treated for 2 h with 10 μM CCCP and imaged in Figure 4C. Data are represented as mean ± SEM. Two-tailed unpaired Student's t test, ^∗^p < 0.05, n = 18 from three independent experiments. (E) HA-Parkin-expressing HEK293 cells were treated for 2 h with 10 μM CCCP alone or with the addition of 100 μM CK666. After washout, cells were fixed every hour for 3 h, stained with Cyt c to visualize mitochondria and DAPI to label nuclei, and imaged by widefield microscopy. Images were scored according to three categories of mitochondrial morphology: clustered, refusing, and network (representative images taken by confocal microscopy). Data are represented as means ± SEM. Two-way repeated measures ANOVA with *post-hoc* Bonferroni correction, ^∗^p < 0.05, ^∗∗^p < 0.01, n = 3 (≥531 cells per time point). (F) Schematic model on the role of MYO6 and F-actin: after a mitochondrial insult, the network fragments and Parkin (orange) is selectively recruited to damaged mitochondria, where it ubiquitinates outer mitochondrial membrane proteins. Subsequently, MYO6 (black) is recruited to damaged mitochondria by binding to ubiquitin chains (yellow), and damaged mitochondria are isolated in an F-actin cage (red) from the neighboring population. F-actin cages around damaged mitochondria serve as a barrier to prevent refusion with the mitochondrial network and require MYO6 as well as several actin regulators including cdc42, Arp2/3 complex, formins, and N-WASP.

To test whether MYO6-dependent F-actin cages are important for isolating damaged mitochondria and preventing refusion with neighboring populations, we analyzed the effect of these actin structures on mitochondrial morphology and their ability to fuse with other mitochondria after stress-induced fragmentation. To determine if there were any differences in the size of mitochondria with or without F-actin cages, we quantitated the mitochondrial area in HEK293 cells treated with CCCP for 6 h (Figures 5A and 5B). Mitochondria surrounded by F-actin cages are smaller in size compared with those lacking actin (Figure 5C). On the contrary, in cells overexpressing the dominant-negative MYO6 tail, which inhibits F-actin cage assembly (Figures 4C, 4E, and 4F), mitochondrial fragments were larger in area compared with cells overexpressing full-length MYO6 (Figure 5D).

To confirm whether F-actin cages indeed inhibit refusion of mitochondrial fragments and restrict mitochondrial fragment size, we treated HEK293 cells expressing Parkin with CCCP for 2 h to generate the F-actin cages. In parallel, we inhibited actin cage assembly by treating cells with the Arp2/3 inhibitor, CK666 (Figure 4A). After washout of CCCP, we monitored mitochondrial network reformation in a 3 h time course by widefield microscopy, and quantified the number of cells with either (1) clustered, fragmented mitochondria, (2) refusing mitochondria, or (3) an intact mitochondrial network (Figure 5E). The absence of actin cages caused by CK666 significantly accelerated refusion of fragmented mitochondria, with a concomitant enhanced decrease in the clustered mitochondrial population and increase in cells displaying mitochondrial network morphology (Figure 5E). In summary, these results support our hypothesis that the MYO6-induced F-actin cages trap and isolate mitochondrial fragments by forming a physical barrier around them that limits mitochondrial refusion and network reformation (see our model, Figure 5F).

### Loss of MYO6 Leads to Delayed Clearance of Damaged Mitochondria by Parkin-Mediated Mitophagy

**Figure 6:**
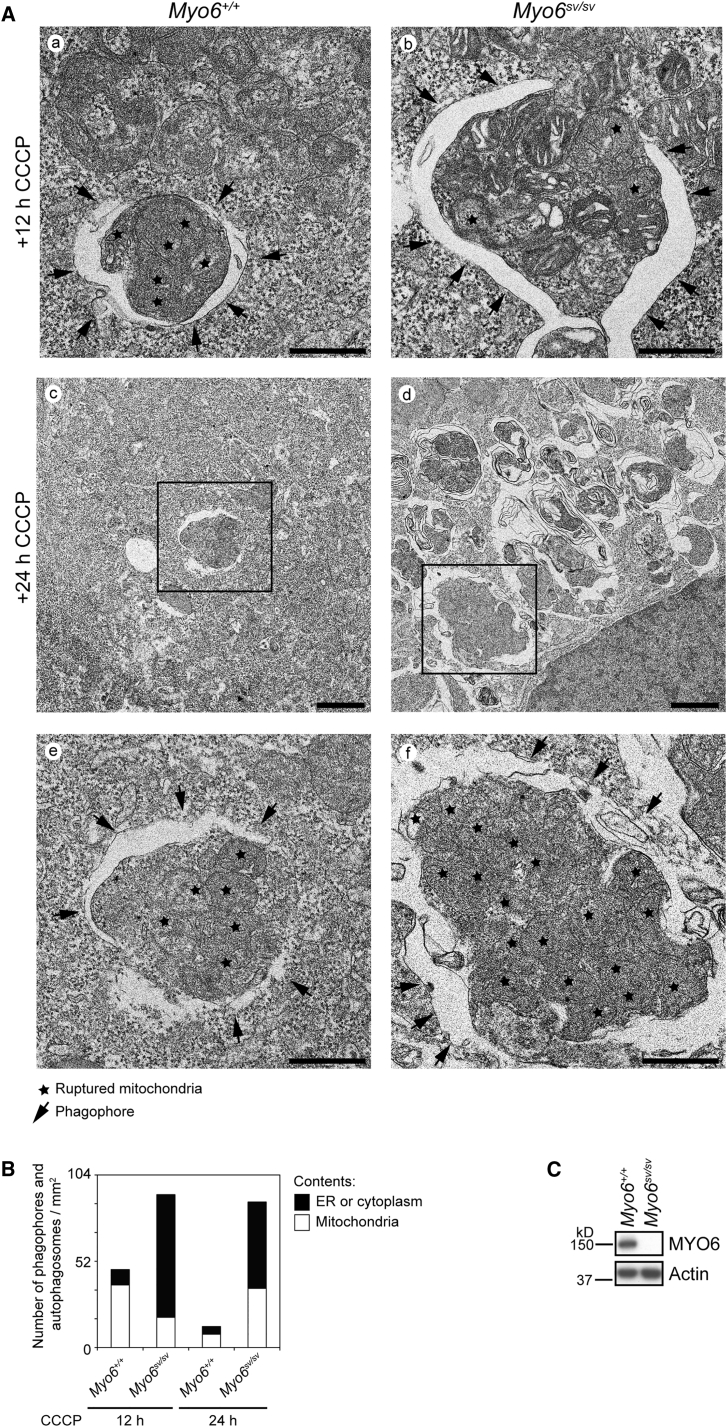
Loss of MYO6 Leads to an Accumulation of Damaged Mitochondria in Autophagosomes after Mitophagy Induction (A) Immortalized wild-type (*Myo6*^*+/+*^) and *Snell's waltzer* (*Myo6*^*sv/sv*^) mouse embryonic fibroblasts (MEFs) were transiently transfected with HA-Parkin and treated for 12 or 24 h with 20 μM CCCP. Cells were imaged by confocal microscopy, processed for correlative light electron microscopy, and imaged by transmission electron microscopy. The star indicates ruptured mitochondria and the arrow shows the phagophore. Scale bars, 500 nm in subpanels (a, b, e, and f), 1 μm in subpanels (c and d). (B) Quantitation of the number of autophagosomes containing mitochondria (white) or endoplasmic reticulum and cytoplasmic material (black) in (A). At least 19 randomly selected areas were counted per cell type. (C) Western blot analysis confirming the absence of MYO6 from *Snell's waltzer* immortalized MEFs compared with wild-type lysates. Actin is shown as a loading control. See also Figure S7.

We next used electron microscopy (EM) to assess the impact of MYO6 depletion on mitochondrial turnover by Parkin-dependent mitophagy. We transiently expressed HA-Parkin in *Snell's waltzer* immortalized mouse embryonic fibroblasts (MEFs) that lack MYO6 (*Myo6*^*sv/sv*^) as well as wild-type controls, and induced mitophagy by CCCP treatment (Figure 6). To ensure that we compared cells with matching expression levels of Parkin, which regulates the rate of mitophagy, we performed correlative light electron microscopy (CLEM). For this procedure, HA-Parkin expression was first visualized by immunofluorescence, and target cells expressing comparable Parkin levels identified by confocal microscopy, which were subsequently analyzed by EM.

In wild-type, and also in *Myo6*^*sv/sv*^ MEFs, we observed the formation of phagophores and autophagosomes containing either endoplasmic reticulum and cytoplasm or mitochondria after 12 h of CCCP treatment indicating no defect in autophagosome formation (Figure 6A). Our quantitation, however, revealed a clear increase in the total number of autophagosomes in *Myo6*^*sv/sv*^ MEFs at 12 h after CCCP treatment, and a higher proportion of autophagosomes containing mitochondria at 24 h after CCCP treatment (Figures 6A, panel d and 6B). This block in mitophagosome clearance confirms our previous results describing an overall accumulation of LC3-positive structures in *Myo6*^*sv/sv*^ MEFs by immunofluorescence and immunoblotting (Tumbarello et al., 2012). Importantly, in contrast to wild-type MEFs where mitophagosomes are degraded after 24 h of CCCP treatment, *Myo6*^*sv/sv*^ cells accumulate autophagosomes containing damaged mitochondria (Figure 6B), implying a kinetic delay in mitophagy.

Taken together, MYO6 is required at two stages of mitophagy: (1) for the recruitment and assembly of F-actin cages around damaged mitochondria to target these damaged organelles for degradation by mitophagy, and (2) for the maturation of mitophagosomes to enable fusion with the lysosome for subsequent degradation.

### Cells Lacking MYO6 Accumulate Functionally Impaired Mitochondria

**Figure 7:**
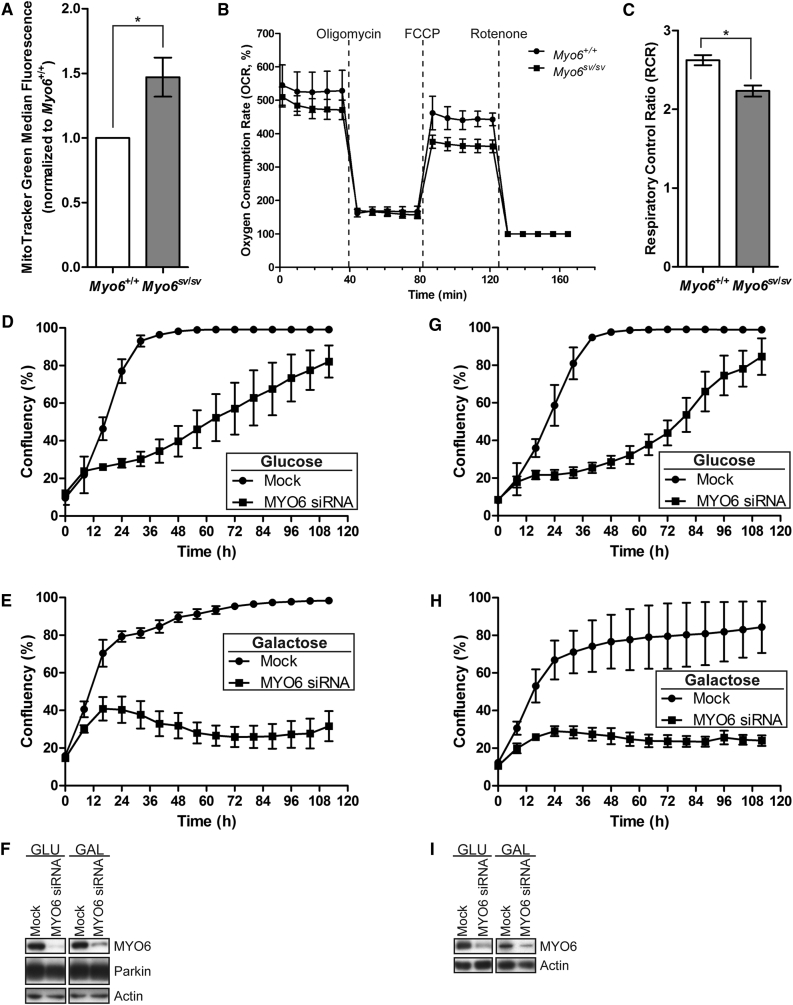
Cells Lacking MYO6 Accumulate Dysfunctional Mitochondria (A) The mitochondrial mass of wild-type (*Myo6*^*+/+*^) and *Snell's waltzer* (*Myo6*^*sv/sv*^) immortalized MEFs was measured using the MitoTracker Green FM dye by flow cytometry. Data are represented as mean ± SEM. Two-tailed paired Student's t test, ^∗^p < 0.05, n = 5. (B) The oxygen consumption rate (OCR), an indicator of mitochondrial respiration, of wild-type and *Snell's waltzer* immortalized MEFs was measured using the XF^e^24 Extracellular Flux Analyser. Oligomycin (1 μM), FCCP (0.75 μM), and rotenone (1 μM) were injected at the indicated times to determine the oxygen consumption rate at regular intervals. Normalized to rotenone as baseline. Data are represented as mean ± SEM, n = 3. (C) Respiratory control ratio ([RCR]; OCR after FCCP addition divided by oligomycin) in wild-type and *Snell's waltzer* immortalized MEFs. Data are represented as mean ± SEM. Two-tailed paired Students t test, ^∗^p < 0.05, n = 3. (D–I) HEK293 cells stably expressing HA-Parkin (D and E) or HEK293 cells (G and H) were depleted of MYO6 by siRNA transfection and growth curves in media containing (D and G) glucose (GLU) or (E and H) galactose (GAL) were obtained by quantitative live-cell phase contrast imaging. Data are represented as mean ± SEM, n = 3. (F) Representative western blot analysis of lysates from (D) and (E) confirming depletion of MYO6 and similar expression levels of Parkin. Actin is shown as a loading control. (I) Representative western blot analysis of lysates from (G) and (H) confirming depletion of MYO6 using actin as a loading control.

To confirm the mitophagy defect in *Myo6*^*sv/sv*^ MEFs, we first measured the total mitochondrial mass by staining with the MitoTracker Green FM dye, which is taken up by mitochondria independent of their membrane potential and therefore labels both healthy and damaged mitochondria. Flow cytometry revealed a 50% increase in mitochondrial mass in *Myo6*^*sv/sv*^ MEFs compared with wild-type cells (Figure 7A), suggesting that they have more mitochondria. We also observed striking morphological changes in the hippocampal cortex of 16-month-old *Snell's waltzer* mice starved overnight, where the mitochondria inside astrocytes appear swollen compared with those of wild-type mice (Figure S7). Although *Myo6*^*sv/sv*^ cells have more mitochondria, these also appear to be more damaged, corroborating our EM results where we observe a defect in the clearance of dysfunctional mitochondria by mitophagy (Figure 6).

To assess the impact that loss of MYO6 has on overall mitochondrial homeostasis, we tested the functionality of mitochondria in wild-type and *Myo6*^*sv/sv*^ MEFs by measuring the oxygen consumption rate, an indicator of mitochondrial respiration (Figure 7B). MYO6-deficient cells had a significantly reduced respiratory control ratio (refer to the STAR Methods), indicating compromised mitochondrial function (Figure 7C). To further evaluate mitochondrial function in the presence and absence of MYO6, we challenged MYO6 siRNA and mock-treated HEK293 cells with exogenous (Figures 7D–7F) or endogenous (Figures 7G–7I) Parkin expression by growing them in medium containing galactose as the sole carbon source, which forces cells to rely on mitochondrial oxidative phosphorylation (OXPHOS) for energy generation. MYO6 knockdown cells displayed a slight growth defect in glucose-containing medium, as previously reported, most likely due to a defect in cytokinesis (Arden et al., 2007); however, they still reached confluency (Figures 7D and 7G). Whereas mock-treated cells grew at almost the same rate in galactose medium as in standard medium with glucose, cells depleted of MYO6 did not grow in galactose medium, indicative of a mitochondrial defect (Figures 7E and 7H). Interestingly, we made similar observations in HEK293 cells with endogenous Parkin (Figures 7G–7I) compared with cells overexpressing Parkin (Figures 7D–7F), indicating that MYO6, but not Parkin, levels are critical to maintain a healthy population of functioning mitochondria to enable cell growth in galactose medium (Figures 7E and 7H).

In short, our results demonstrate that, in the absence of MYO6, mitochondrial function is impaired and dysfunctional mitochondria accumulate, implicating MYO6 as a novel player in mitochondrial quality control and maintenance of mitochondrial homeostasis.

## Discussion

Here, we demonstrate a dual role for MYO6 in mitochondrial quality control. First, we uncover a novel requirement for this motor in organizing Parkin-dependent F-actin cages that isolate damaged mitochondria to limit refusion with neighboring organelles (Figure 5). Second, MYO6 mediates the clearance of damaged mitochondria by Parkin-dependent mitophagy via its associations with the mitophagy receptors, in particular the maturation of mitophagosomes and fusion with lysosomes (Tumbarello et al., 2012). Loss of MYO6 results in functionally impaired mitochondria that have a reduced respiratory capacity, and MYO6 knockdown cells failed to grow when forced to rely on mitochondrial OXPHOS (Figure 7). Although cells lacking MYO6 have more mitochondrial mass, these mitochondria appear damaged, which we observe as swollen mitochondria in the astrocytes of the hippocampal cortex of starved *Snell's waltzer* mouse brains (Figure S7), in agreement with the published literature (Osterweil et al., 2005). Thus, we demonstrate that the absence of MYO6 leads to mitochondrial dysfunction. Interestingly, some phenotypes of the MYO6 KO mouse, such as hypertrophic cardiomyopathy and gliosis in the brain (Mohiddin et al., 2004, Osterweil et al., 2005), are reminiscent of mitochondrial diseases (Chinnery, 2015) and neurodegenerative disorders, respectively.

We show that MYO6 recruitment to damaged mitochondria after mitophagy induction and in the presence of Parkin does not involve the mitophagy receptors. Instead, this motor is directly targeted to mitochondria after OA or CCCP treatment by binding to ubiquitin via its MyUb/RRL domain (Figures 1, 2, S1A, and S1B). This ubiquitin-binding domain also encompasses the RRL motif (He et al., 2016), which enables direct binding to the mitophagy receptors (TAX1BP1, NDP52, and OPTN) (Morriswood et al., 2007, Sahlender et al., 2005). Intriguingly, the MYO6 and ubiquitin-binding sites also overlap in TAX1BP1, NDP52, and OPTN (Morriswood et al., 2007, Tumbarello et al., 2015), suggesting that binding of MYO6 to the autophagy receptors or to ubiquitin is mutually exclusive. These findings imply that MYO6 and the mitophagy receptors are recruited independently and in parallel to damaged mitochondria. This model is supported by our results showing that translocation of TAX1BP1, NDP52, or OPTN is not impaired in MYO6 KO cells generated by CRISPR/Cas9 genome editing, and that MYO6 is still recruited to damaged mitochondria in cells depleted of all three mitophagy receptors (Figures 2C and S4).

The recruitment of MYO6 via the MyUb/RRL domain is strictly dependent on the presence of Parkin and its E3 ligase activity (Figure S1C) that decorates the surface of mitochondria with K63, K48, K6, and K11 ubiquitin chains (Cunningham et al., 2015, Ordureau et al., 2014). Indeed, our *in vitro* studies demonstrate that MYO6 can bind directly via the MyUb/RRL domain to K63 and K11 tetramers, as shown previously for di-ubiquitin (He et al., 2016), a similar ubiquitin chain preference as TAX1BP1 (Figure S4). Our *in vitro* competition assay demonstrates that increasing amounts of TAX1BP1 bound to ubiquitin tetramers, but did not displace MYO6, thus further highlighting the parallel and independent recruitment of MYO6 and TAX1BP1 to damaged mitochondria.

MYO6 is also in a complex with Parkin, as both proteins can be co-immunoprecipitated in the presence or absence of CCCP (Figure 1), thereby refining results from a large-scale interaction proteomics study (Sarraf et al., 2013). Our data seem to suggest that the MYO6-Parkin interaction is not dependent on recruitment to mitochondria, but may also take place in other cellular compartments. Indeed, the roles of Parkin are not restricted to mitophagy, but also include regulation of cytokine secretion and cell surface receptor endocytosis (de Léséleuc et al., 2013, Fallon et al., 2006).

After CCCP treatment to induce mitochondrial damage, we observe two distinct waves of F-actin assembly on mitochondria (Figure 3). In agreement with previous studies, we show that the dynamic and transient actin filament polymerization on the OMM happens within 5 min of CCCP treatment, which is required for mitochondrial fission (Korobova et al., 2013, Li et al., 2015). Myosin II, which can assemble into bipolar filaments, is enriched at these sites and is thought to provide the force for constriction together with actin polymerization as inhibition of myosin II activity leads an increase in mitochondrial length (Hatch et al., 2014, Korobova et al., 2014). However, MYO6 is not recruited at this time point to mitochondria and is not required for mitochondrial fission (Figures 3 and S5C).

In contrast to this immediate and short-lived actin response for mitochondrial fission, we observe a MYO6- and Parkin-dependent assembly of stable F-actin cages around fragmented mitochondria a few hours after mitochondrial damage induction with CCCP, which corresponds to the spatiotemporal recruitment of MYO6 to damaged mitochondria (Figures 3, 4, and S5). The formation of these actin structures on damaged mitochondria is strongly inhibited in cells expressing the dominant-negative MYO6 tail domain, indicating that full-length MYO6 containing the motor domain is required for the organization of F-actin cages around damaged mitochondria (Figure 4). A role for this myosin in regulating actin filament dynamics and assembly has been reported previously; for example, MYO6 has the capacity to promote the local accumulation of actin structures at cell-cell contacts, such as the zonula adherens of polarized epithelial cells (Mangold et al., 2011), and in actin cones of segregating cells during *Drosophila* spermatid individualization (Noguchi et al., 2006). Although the exact mechanism of MYO6-dependent actin regulation is currently not known, MYO6 has been identified in a complex with DOCK7, a guanine nucleotide exchange factor for Rac and cdc42, which performs important functions in the spatial regulation of actin organization (Majewski et al., 2012, Sobczak et al., 2016). In addition, we demonstrate a strong requirement for cdc42, Arp2/3, formins, and N-WASP in F-actin cage formation around damaged mitochondria (Figure 4), suggesting tight control of actin filament assembly around mitochondria.

Finally, we provide mechanistic insight into the functional requirement of the F-actin cages recruited by MYO6. Loss of these cages by overexpression of dominant-negative tails (Figure 4) results in larger mitochondrial fragment size, and treatment with the Arp2/3 inhibitor to prevent cage formation leads to an increased rate of refusion after CCCP washout (Figure 5). Thus, these MYO6-dependent actin cages isolate and quarantine damaged mitochondria by forming a physical barrier to inhibit refusion, and potentially content exchange, of damaged subpopulations destined for mitophagy with neighboring organelles. This actin-caging mechanism may therefore be a critical quality control step to ensure mitochondrial homeostatic regulation.

### Conclusions

We have identified essential roles for MYO6 in mitochondrial turnover and homeostasis. The loss of MYO6 leads to reduced clearance of autophagosomes containing mitochondria, and causes the accumulation of damaged mitochondria with impaired respiratory capacity. Cells without MYO6 are no longer able to grow in galactose medium, which requires fully functional mitochondria for energy production via OXPHOS. We propose a dual role for MYO6 in Parkin-dependent mitophagy. Firstly, MYO6 is recruited via ubiquitin directly to mitochondria, where it induces the formation of F-actin cages, which serve as a quality control mechanism to quarantine damaged mitochondria and prevent refusion with neighboring populations after mitophagy induction (Figure 5F). Secondly, the maturation of autophagosomes containing mitochondria to enable lysosomal degradation is mediated by MYO6, in agreement with our previous model (Tumbarello et al., 2012, Tumbarello et al., 2013).

## STAR★Methods

### Key Resources Table

**Table:** 

REAGENT or RESOURCE	SOURCE	IDENTIFIER
**Antibodies**		

Rabbit polyclonal to actin	Sigma-Aldrich	Cat# A2066; RRID AB_476693
Mouse monoclonal to cytochrome c, clone 6H2.B4	BioLegend	Cat# 612302; RRID AB_315775
Rabbit polyclonal to DsRed	Clontech Laboratories	Cat# 632496; RRID AB_10013483
Mouse monoclonal to GFP, clone 9F9.F9	Abcam	Cat# ab1218; RRID AB_298911
Rabbit polyclonal to GFP	Thermo Fischer Scientific	Cat# A-11122; RRID AB_221569
Rabbit polyclonal to GFP	Abcam	Cat# ab6556; RRID AB_305564
Rabbit polyclonal to GST	Sigma-Aldrich	Cat# G7781; RRID AB_259965
Mouse monoclonal to HA, clone HA-7	Sigma-Aldrich	Cat# H9658; RRID AB_260092
Rat monoclonal to HA, clone 3F10	Roche	Cat# 11867423001; RRID AB_390918
Rabbit polyclonal to optineurin (Ab2)	Sigma-Aldrich	Cat# HPA003360; RRID AB_1079528
Mouse monoclonal to parkin, clone PRK8	Santa Cruz Biotechnology	Cat# sc-32282; RRID AB_628104
Rabbit polyclonal to Tom20 (FL-145)	Santa Cruz Biotechnology	Cat# sc-11415; RRID AB_2207533
Mouse monoclonal to α-tubulin, clone DM1A	Sigma-Aldrich	Cat# T9026; RRID: AB_477593
Mouse monoclonal to ubiquitin, clone FK2	Millipore	Cat# ST1200; RRID: AB_10681625
Affinity-purified rabbit polyclonal to MYO6	(Buss et al., 1998)	N/A
Affinity-purified rabbit polyclonal to TAX1BP1	(Morriswood et al., 2007)	N/A
Affinity-purified rabbit polyclonal to NDP52	(Morriswood et al., 2007)	N/A
Affinity-purified rabbit polyclonal to OPTN	(Sahlender et al., 2005)	N/A

**Bacterial and Virus Strains**		

HA-Parkin/pMXs-IP	(Yoshii et al., 2011)	Addgene; Cat# 38248
HA-Parkin C431S/pMXs-IP	This paper	N/A

**Chemicals, Peptides, and Recombinant Proteins**		

Carbonyl cyanide 3-chlorophenylhydrazone (CCCP)	Fischer Scientific	Cat# AC228131000
Oligomycin	Millipore	Cat# 495455
Antimycin A	Sigma-Aldrich	Cat# A8674
K63 tetraubiquitin (Ub^4^)	Boston Biochem	Cat# UC-310
K11 Ub^4^	(Grice et al., 2015)	N/A
Rhosin	Calbiochem	Cat# 555460
Rho inhibitor I	Cytoskeleton	Cat# CT04
NSC23766 trihydrochloride	Sigma-Aldrich	Cat# SML0952
EHT 1864	Tocris Bioscience	Cat# 3872
W56	Tocris Bioscience	Cat# 2221
ML141	Sigma-Aldrich	Cat# SML0407
CK666	Abcam	Cat# ab141231
Formin FH2 Domain Inhibitor (SMIFH2)	Calbiochem	Cat# 344092
Wiskostatin	Sigma-Aldrich	Cat# W2270

**Critical Commercial Assays**		

Seahorse XF Cell Mito Stress Test Kit	Agilent Technologies	Cat# 103015-100

**Experimental Models: Cell Lines**		

Human HEK293	ECACC	Cat# 85120602
HA-Parkin expressing HEK293	This paper	HA-Parkin/HEK293
HA-Parkin C431S expressing HEK293	This paper	HA-Parkin C431S/HEK293
Human HeLaM	Roger Tsien, University of California San Diego	N/A
HA-Parkin expressing HeLaM	This paper	HA-Parkin/HeLaM
CRISPR/Cas9 MYO6 knockout HeLaM	(Brooks et al., 2017)	N/A
Wild-type (*Myo6*^+/+^) immortalized MEF	This paper	N/A
*Snell’s waltzer* (*Myo6*^*sv/sv*^) immortalized MEF	This paper	N/A

**Experimental Models: Organisms/Strains**		

Mouse: C57BL/6 wild-type (*Myo*^*+/+*^) and *Snell’s waltzer* (*Myo6*^*sv/sv*^)	(Avraham et al., 1995)	N/A

**Oligonucleotides**		

SDM primers for HA-Parkin C431S/pMXs-IP: Parkin_C431S_For GAAAAAAATGGAGGCAGCATGCACATGAAG Parkin_C431S_Rev CTTCATGTGCATGCTGCCTCCATTTTTTTC	This paper	N/A
SDM primers for hMYO6 FL I1104A/pEGFP-C3: MYO6_I1104A_For ATCAATACTTCTTGTGATG CTGAGCTCCTGGCAG MYO6_I1104A_Rev CTGCCAGGAGCTCAGCA TCACAAGAAGTATTGAT	This paper	N/A
SDM primers for hMYO6 FL D179Y/pEGFP-C3: MYO6_D179Y_For GGAACAGGTCAAGATATTT ATGACAGAATTGTTGAAGC MYO6_D179Y_Rev GCTTCAACAATTCTGTCATAAATATCTTGACC TGTTCC	This paper	N/A
PCR primers for hMYO6 CBD/pRSET-A: MYO6_CBD_For GAGCTCGAGCTGCGGAGA GGTCCTGCTGTA MYO6_CBD_Rev GCCGAATTCCTACTTTAAC AGACTCTGCAGCAT	This paper	N/A
Mouse genotyping primers: ex18F TGAAGTCTGACCCTGATCACTT ex19R GCAAAGCCACATTCGGATGG	This paper	N/A

**Recombinant DNA**		

Parkin WT/pEGFP-C2	(Trempe et al., 2013)	Addgene; Cat# 45875
Parkin/mCherry-C1	(Narendra et al., 2008)	Addgene; Cat# 23956
pEF321-T	Sumio Sugano, University of Tokyo	N/A
Full-length (FL) human (h) MYO6 (isoform 5 containing no alternative splicing inserts, UniProtKB: Q9UM54-5) wild type (WT)/pEGFP-C3	(Bond et al., 2012)	GFP-MYO6 FL
hMYO6 FL K175R^∗^ (rigor)/pEGFP-C3 ^∗^ all amino acid designations are according to the canonical human MYO6 sequence (isoform 3, UniProtKB: Q9UM54-3)	(Arden et al., 2016)	GFP-MYO6 FL K175R
hMYO6 FL A1013G (MIU)/pEGFP-C3	(Tumbarello et al., 2012)	GFP-MYO6 FL A1013G
hMYO6 FL R1116A/R1117A/L1118A (RRL)/pEGFP-C3	(Arden et al., 2016)	GFP-MYO6 FL RRL
hMYO6 FL W1202L (WWY)/pEGFP-C3	(Arden et al., 2016)	GFP-MYO6 FL WWY
hMYO6 tail/pEGFP-C3	(Chibalina et al., 2010)	GFP-MYO6 tail
hMYO6 FL I1104A (MyUb)/pEGFP-C3	This paper	GFP-MYO6 FL I1104A
hMYO6 FL D179Y (tailchaser)/pEGFP-C3	This paper	GFP-MYO6 FL D179Y
hTAX1BP1 (isoform 2, UniProtKB: Q86VP1-2; aa 425–789^§^)/pRSET-A ^§^ all amino acid designations are according to the canonical TAX1BP1 sequence (isoform 1, UniProtKB Q86VP1-1)	(Morriswood et al., 2007)	His-TAX1BP1 (C-terminal half)
hMYO6 cargo binding domain (CBD, aa 1034–1294)/pGEX-4T1	(Arden et al., 2016)	GST-MYO6 CBD
hMYO6 CBD/pRSET-A	This paper	His-MYO6 CBD

**Software and Algorithms**		

Prism	GraphPad	N/A
ZEN Blue, ZEN Black, ZEN Black ELYRA	Carl Zeiss Microscopy	N/A
Volocity	PerkinElmer	N/A
Fiji	NIH	N/A
FlowJo	BD	N/A
Seahorse XF^e^ Wave	Agilent Technologies	N/A
IncuCyte 2011A	Essen Bioscience	N/A
Photoshop, InDesign, Illustrator	Adobe	N/A

### Contact for Reagent and Resource Sharing

Further information and requests for resources and reagents should be directed to and will be fulfilled by the Lead Contact, Folma Buss (fb207@cam.ac.uk). MTAs were obtained for all Addgene constructs.

### Experimental Model and Subject Details

All cells were incubated at 37°C and 5% CO_2_.

#### HEK293 Cell Culture

HEK293 cells were grown in MEM (M2279, Sigma-Aldrich) supplemented with 10% fetal bovine serum (FBS, F7524, Sigma-Aldrich), 1% non-essential amino acids (M7145, Sigma-Aldrich), 2 mM L-glutamine (G7513, Sigma-Aldrich), 100 U/ml penicillin and 100 μg/ml streptomycin (P4333, Sigma-Aldrich).

#### HeLaM Cell Culture

HeLaM and CRISPR/Cas9 MYO6 knockout HeLaM (Brooks et al., 2017) were cultured in RPMI-1640 (R8758, Sigma-Aldrich) containing 10% FBS, 100 U/ml penicillin and 100 μg/ml streptomycin. Immortalized MEFs were maintained in DMEM (31966-021, Thermo Fischer Scientific) supplemented with 10% FBS, 2 mM L-glutamine, 100 U/ml penicillin and 100 μg/ml streptomycin.

#### Snell’s Waltzer Mice

The *Snell’s waltzer* mice (C57BL/6 background) were bred and housed under pathogen-free conditions in the animal facility at Cambridge University. Experimentation involving animals was carried out under a UK Home Office Project Licence granted to Dr Folma Buss (PPL 70/8460) and was approved by the UK Home Office and the University of Cambridge Animal Welfare and Ethical Review Committee. The work has been carried out in accordance to the UK Animals (Scientific Procedures) Act 1986 and follows the Laboratory Animal Science Association (LASA) Guidelines.

### Method Details

#### Antibodies

The antibodies listed in the Key Resources Table were used as follows: rabbit polyclonal antibody to actin (A2066, Sigma-Aldrich, WB 1:2,000), mouse monoclonal to cytochrome c (6H2.B4, 612302, BioLegend, IF 1:200), rabbit polyclonal to DsRed (632496, Clontech Laboratories, IF 1:500, WB 1:1,000), mouse monoclonal to GFP (9F9.F9, ab1218, abcam, IF 1:400–1,000), rabbit polyclonal to GFP (A11122, Thermo Fischer Scientific, IF 1:400), rabbit polyclonal to GFP (ab6556, abcam, WB 1:1,000), rabbit polyclonal to GST (G7781, Sigma-Aldrich, WB 1:20,000), mouse monoclonal to HA (HA-7, H9658, Sigma-Aldrich, IP), rat monoclonal to HA (3F10, 11867423001, Roche, IF 1:100, IP), rabbit polyclonal to optineurin (Ab2, HPA003360, Sigma-Aldrich, IF 1:100), mouse monoclonal to parkin (PRK8, sc-32282, Santa Cruz Biotechnology, WB 1:500), rabbit polyclonal to Tom20 (FL-145, sc-11415, Santa Cruz Biotechnology, IF 1:100), mouse monoclonal to α-tubulin (DM1A, T9025, WB 1:2,000), mouse monoclonal to ubiquitin (P4D1, sc-8017, Santa Cruz Biotechnology, WB 1:1,000), mouse monoclonal to ubiquitin (FK2, ST1200, Millipore, WB 1:1,000). Affinity-purified rabbit polyclonal antibodies against MYO6, TAX1BP1, NDP52, and OPTN (IF 1:100, WB 1:1,000) were generated as previously described (Buss et al., 1998, Morriswood et al., 2007, Sahlender et al., 2005).

#### Plasmids

The construct to express catalytically inactive HA-Parkin harboring the C431S mutation in cells was generated by site-directed mutagenesis (SDM) of the HA-Parkin/pMXs-IP construct using the Parkin_C431S_For and Parkin_C431S_Rev primers listed in the Key Resources Table. By SDM of full-length (FL) human (h) MYO6 in the pEGFP-C3 vector, the MyUb mutant (I1104A) was generated using MYO6_I1104A_For and MYO6_I1104A_Rev primers and the tailchaser mutant (D179Y) using the MYO6_D179Y_For and MYO6_D179Y_Rev primers listed in the Key Resources Table. Human MYO6 CBD (aa 1034–1294) was amplified by PCR using hMYO6 FL/pEGFP-C3 as a template with the MYO6_CBD_For and MYO6_CBD_Rev primers and cloned into pRSET-A using *Xho*I/*Eco*RI sites. All constructs were verified by sequencing (Source BioScience).

#### Transfections

Transient DNA transfections were performed using FuGENE 6 (Promega) according to the manufacturer’s instructions. For efficient knockdown, cells were transfected twice with ON-TARGET*plus* SMARTpool siRNA oligonucleotides (Dharmacon, GE) against human MYO6, TAX1BP1, NDP52, or OPTN using Oligofectamine (Thermo Fischer Scientific) on day 1 and 3. For the mock transfection, the siRNA was replaced with Opti-MEM (Thermo Fischer Scientific). If required, cells were transfected with DNA constructs on day 4. On day 5, the cells were processed for the corresponding assay and the efficiency of protein depletion assessed by Western blotting.

#### Generation of Stable Cell Lines

HEK293 and HeLaM cells stably overexpressing HA-Parkin WT or HA-Parkin C431S were generated by retroviral transduction of the HA-Parkin WT or C431S constructs in the pMXs-IP vector. Single-cell clones were selected with 1 μg/ml puromycin (Thermo Fischer Scientific) and these stable cell lines were maintained in HEK293 or HeLaM medium supplemented with 1 μg/ml puromycin.

#### Mouse Genotyping and Establishment of Immortalized Mouse Embryonic Fibroblasts

Wild-type and *Snell’s waltzer* C57BL/6J mice (Avraham et al., 1995) were genotyped using a PCR-based method (Self et al., 1999) with modifications. Genomic DNA from tails was isolated using the High Pure PCR Template Preparation Kit (Roche) and amplified with ex18F and ex19R primers listed in the Key Resources Table that flank the deletion in the *sv* allele. Wild-type (*Myo*^*+/+*^) mice displayed a genomic fragment of 2.3 kb and *Snell’s waltzer* (*Myo6*^*sv/sv*^) mice displayed a 1.3 kb fragment, while heterozygous mice generated both fragments. Primary embryonic fibroblasts from wild-type and *Snell’s waltzer* mice were prepared as previously described (Warner et al., 2003) and immortalized with the SV40 large T-antigen (pEF321-T) by transfection with Lipofectamine 2000 (Thermo Fischer Scientific). The media was changed after 24 hours (h) and cells were expanded over several weeks to make mixed immortalized MEF cultures.

#### Western Blot and Co-Immunoprecipitation

Cell lysates were prepared in ice-cold RIPA lysis buffer (1% Triton, 0.1% SDS, 1% DOC, 150 mM NaCl, 10 mM Tris, pH 7.4, 5 mM EDTA) containing protease inhibitors (cOmplete mini, EDTA-free, Roche), boiled in SDS loading buffer, and separated by SDS-PAGE. After protein transfer on to PVDF (Immobilon-P, Millipore), the membrane was blocked with 5% fat-free milk powder in PBS-T (0.05% Tween-20 in PBS) for 1 h at room temperature (RT) and incubated with primary antibodies overnight at 4°C. The membranes were washed three times with PBS-T and incubated with the corresponding HRP-conjugated secondary antibody for 1 h at RT. After washing as before, the protein bands were detected with ECL or ECL Prime Western blotting detection reagent (GE Healthcare Life Sciences) according to the manufacturer’s protocol and exposed to X-ray film (Fujifilm).

Co-immunoprecipitation experiments were performed from HA-Parkin WT/HEK293 stable cell lines under native conditions. Cells were collected in ice-cold native lysis buffer [50 mM Tris, pH 7.4, 100 mM NaCl, 1% NP-40, 5 mM MgCl_2_, 5 mM ATP (Roche) supplemented with protease inhibitors (cOmplete mini, EDTA-free, Roche), phosphatase inhibitors (PhosSTOP, Roche), 1 mM PMSF (Viva Bioscience), 1 mM NEM, 1 mM IAA] and cell debris pelleted at 20000 ×g for 15 min. The supernatants were precleared with either Protein A or G sepharose beads for 1 h, incubated with 5 μg antibody for 1 h, and subsequently with Protein A or G sepharose beads for 1 h at 4°C with end-over-end mixing. The beads were washed four times with 0.2% NP-40 in TBS, once with TBS, and protein complexes were eluted off the beads with 5X SDS loading buffer before separation by SDS-PAGE and immunoblotting.

#### Immunofluorescence and Confocal/Widefield Microscopy

Cells plated on glass coverslips were washed with PBS and fixed with 4% formaldehyde (Electron Microscopy Sciences) for 20 min, washed three times with PBS, and permeabilized with 0.2% Triton X-100 in PBS for 2 min. After blocking in 1% BSA in PBS for 30 min, cells were incubated with primary antibodies for 1 h at RT in a humidified chamber. After three washes with PBS and incubation with the appropriate AlexaFluor 488-, 568-, 647-conjugated species-specific secondary antibodies (1:300, Thermo Fischer Scientific) or AlexaFluor 488/568-conjugated phalloidin (1:1000, Thermo Fischer Scientific) for 45 min, coverslips were mounted with ProLong Gold Antifade (Thermo Fischer Scientific). Images were acquired on LSM 710 or LSM 880 confocal microscopes (Carl Zeiss Microscopy) with ZEN Black (Carl Zeiss Microscopy) software or an AxioImager Z2 widefield microscope with ZEN Blue (Carl Zeiss Microscopy) software.

#### Superresolution Structured Illumination Microscopy (SR-SIM)

Acid-washed high performance coverslips (#1.5H, thickness 170±5 μm, Schott) were used, processed for immunofluorescence as above with the exception of using eight times the concentration of fluorescently labelled phalloidin (1:50) and mounting on unfrosted glass microscope slides (Thermo Fischer Scientific) followed by curing for three days at room temperature in the dark prior to imaging. Z-stacks were acquired at five phases and five rotations of the illumination grid on an ELYRA PS.1 superresolution microscope (Carl Zeiss Microscopy). The images were processed and channel aligned using ZEN Black ELYRA edition (Carl Zeiss Microscopy). Line profiles on single slices were generated in ZEN Black and normalized for each channel.

#### Electron Microscopy (EM)

For correlative light and electron microscopy (CLEM), MEFs transiently expressing HA-Parkin were cultured on glass bottom dishes with a grid pattern (P35G-2-14-C-GRID, MatTek) and treated with 20 μM CCCP for 12 h or 24 h (Kishi-Itakura and Buss, 2017). The cells were fixed with 4% formaldehyde (F017, TAAB) in 0.1 M sodium phosphate buffer (PB, pH 7.4) for 2 h, washed with the same buffer three times, and incubated for 15 seconds (s) in PB containing 14% glycerol and 35% sucrose. After permeabilization by freezing and thawing in liquid nitrogen for 15 s, cells were immunostained with anti-HA antibody followed by Alexa Fluor 488 anti-rat secondary antibody as well as Hoechst and examined under a LSM 780 confocal microscope. The same specimens were further incubated with 2% formaldehyde and 2.5% glutaraldehyde (G011/2, TAAB) in 0.1 M PB for 2 h. After three washes in 0.1 M PB, the samples were post-fixed with 1.5% osmium tetroxide in 0.1 M PB for 2 h, dehydrated in ethanol, and embedded in Agar 100 mixture (Agar Scientific). Ultrathin sections (70 nm thick) were stained with saturated uranyl acetate and lead citrate solution and observed under a FEI Tecnai Spirit transmission electron microscopy (TEM). Images were recorded with a Gatan CCD camera (Gatan US 1000X-U Camera 2000 kV).

For conventional EM analysis of mouse brains, 16-month old mice were starved overnight and fixed by cardiac perfusion using Ringer’s buffer and then 2% glutaraldehyde and 2% formaldehyde in 30 mM HEPES (pH 7.4) buffer as previously described (Takamura et al., 2011). Subsequently, the mouse brains were embedded in 5% low melting point agarose (A4018) in 0.1 M PB and glued to the sectioning block of a vibratome. The sections (0.3–0.4 mm thick) were post-fixed with 1.5% osmium tetroxide in 0.1 M PB for 2 h. Tissues were dehydrated in a graded series of ethanol and embedded in Agar 100 mixture. Ultrathin sections were processed for EM analysis as above.

#### Flow Cytometry

For mitochondrial mass measurements, wild-type (*Myo6*^*+/+*^) and *Snell’s waltzer* (*Myo6*^*sv/sv*^) immortalized MEFs were seeded at 200,000 cells/well and after 48 h, were incubated with 100 nM MitoTracker Green FM dye (Thermo Fischer Scientific) for 45 min at 37°C and 5% CO_2_. Cells were collected by trypsinization, washed and resuspended in PBS, and analyzed immediately by flow cytometry using 488 nm laser (excitation) with 530/30 nm band pass filter (emission) on a BD LSRFortessa. The data was analysed using FlowJo software (vX.0.7) where debris and apoptotic cells were excluded using forward and side scatter gating.

#### Measurement of Mitochondrial Respiration

Wild-type (*Myo6*^*+/+*^) and *Snell’s waltzer* (*Myo6*^*sv/sv*^) immortalized MEFs were seeded in quintuplicate at 40,000 cells/well in 100 μl growth medium in Seahorse XF24 cell culture microplates (Agilent Technologies) and incubated for 24 h at 37°C and 5% CO_2_. One hour before the assay, growth medium was removed, replaced with 630 μl assay medium [XF assay medium (102352-000, Seahorse Bioscience), pH 7.4, supplemented with 5 mM D(+)-galactose (G5388) and 1 mM sodium pyruvate (11360-039, Thermo Fischer Scientific)], and left to stabilize in a 37°C incubator without CO_2_. The wells containing cells were sequentially injected with 70 μl of 10 μM oligomycin (final: 1 μM) to inhibit ATP synthase, 8.25 μM FCCP (final: 0.75 μM) to uncouple the respiratory chain, 12 μM rotenone (final: 1 μM; all inhibitors were part of the Seahorse XF Cell Mito Stress Test Kit, Agilent Technologies) to inhibit complex I and the oxygen consumption rate (OCR) was measured every 5 min using an XF^e^24 Extracellular Flux Analyzer with XF^e^ Wave software (Seahorse Bioscience) (Rorbach et al., 2012). The Respiratory Control Ratio (RCR) was calculated by dividing the OCR after FCCP injection by that after oligomycin addition.

#### Growth Curves by Quantitative Live-Cell Phase Contrast Imaging

HEK293 or HA-Parkin expressing HEK293 cells were grown in either DMEM (31966-021, Thermo Fischer Scientific) containing 10% FBS, 100 U/ml penicillin, and 100 μg/ml streptomycin or DMEM (11966-025, Thermo Fischer Scientific) containing 10% FBS, 1 mM sodium pyruvate, 10 mM D(+)-galactose, 100 U/ml penicillin, and 100 μg/ml streptomycin during the knockdown procedure (see above). On day 3, cells were seeded in a 6-well plate at 150,000 cells/well in glucose medium or 250,000 cells/well in galactose medium and reverse transfected with siRNA against MYO6. Growth curves were obtained using the IncuCyte HD live-cell imaging system (Essen Bioscience), which photographed cells in phase contrast every 8 h for >4 days (Rorbach et al., 2014), and analysed with the IncuCyte 2011A software (Essen Bioscience).

#### Protein Expression and Purification

K11 tetraubiquitin (Ub^4^) was synthesized using the E2 enzyme Ube2S as described previously (Grice et al., 2015). Human TAX1BP1 (aa 425–789) was cloned into the pRSET-A vector (Morriswood et al., 2007) and the His-tagged protein expressed in *E. coli* C41 (DE3) cells. The bacteria were initially grown at 37°C for 5 h (until an optical density of 1.0 at A_600 nm_), then cooled to 20°C, induced with 1 mM isopropyl β-D-1 thiogalactopyranoside (IPTG, Melford) and then grown at 20°C for a further 17–24 h. After cooling, the bacteria were pelleted by centrifugation at 2,500 × g for 30 min. The cell pellets were frozen in liquid nitrogen, thawed, resuspended in lysis buffer (PBS with 200 mM NaCl, 50 mM imidazole, 15 mM β-mercaptoethanol), sonicated and centrifuged at 60000 × g for 30 min. The lysis supernatant was added to equilibrated Ni-NTA agarose resin (Qiagen), washed extensively with lysis buffer and the His-tagged hTAX1BP1 protein eluted using PBS with 200 mM NaCl, 300 mM imidazole pH 7.4, and 15 mM β-mercaptoethanol. Fractions were analysed by SDS-PAGE, pooled and dialyzed against PBS with 200 mM NaCl and 1 mM DTT. His-tagged hMYO6 CBD (aa 1034–1294) was prepared as described previously (Spudich et al., 2007) with the following modifications after application to the Ni-NTA column of washing with PBS containing 100 mM imidazole at pH 7.4 and eluting with PBS containing 300 mM imidazole at pH 7.4. GST-tagged hMYO6 CBD (aa 1034–1294) was prepared as described for GST-tagged MYO6 fusion proteins (Buss et al., 1998) and purified on glutathione sepharose 4B (GE Healthcare) according to the manufacturer’s instructions.

#### Ubiquitin Binding and Competition Assays

His-MYO6 CBD (500 nM) or His-TAX1BP1 (500 nM) were bound to Ni-NTA agarose (Qiagen) in Tris binding buffer (25 mM Tris-HCl, pH 7.4, 250 mM NaCl, 0.1% Triton, 1 mM DTT, BSA 0.25 mg/ml). Increasing concentrations of K63 or K11 Ub^4^ chains were added (25, 50 or 100 nM) and the samples incubated at 4°C for 30 min on a rotator. The resins were washed five times with Tris binding buffer and proteins bound visualised by Western blotting. Competition binding assays were performed by first incubating the GST-MYO6 CBD (300 nM) and K63 Ub^4^ (150 nM) with glutathione sepharose 4B in Tris binding buffer at 4°C for 30 min. The resins were then washed five times before incubation with increasing concentrations of His-TAX1BP1 (150 nM, 300 nM, 600 M, 1.2 μM or 2.4 μM) for 30 min at 4°C. Finally, the resins were washed five times with Tris binding buffer and proteins bound visualised by Western blotting.

#### Presentation of Data

All graphs were produced using Prism (GraphPad) software. Images were processed in Photoshop (Adobe) and assembled into figures using InDesign or Illustrator (Adobe).

### Quantification and Statistical Analysis

#### Image Analysis

For co-localization analysis of confocal images, the Pearson’s correlation coefficient automatically thresholded using the Costes et al. method (Costes et al., 2004) was quantified using Volocity software v6.3 (PerkinElmer). To quantitate the percentage of cells with MYO6 or F-actin on mitochondria, widefield images were scored for the presence or absence of MYO6 or actin on mitochondria. For the mitochondrial morphology quantitation, widefield images were scored according to three categories: clustered, refusing, and network. For quantitation of SR-SIM images, 5 μm^2^ region of interests for mitochondria (with or without F-actin cages) were chosen and the mitochondrial area was determined using the ‘analyse particles’ function with a minimum area of 0.05 μm^2^ in Fiji (NIH). For EM quantitation, cell areas (57.46 μm^2^) were randomly selected on ×2900 zoom and the number of phagophores and autophagosomes containing either mitochondria or ER/cytoplasm were counted (Yoshii et al., 2011).

#### Statistics

Statistics were calculated using a Student’s t test and one- or two-way analysis of variance (ANOVA) followed by a Bonferroni multiple-comparison post-hoc test. In each figure legend, the statistical parameters are stated: how the data is represented (mean ± S.E.M), the number of independent experiments (n≥3), and the statistical test used to obtain *p* values and determine significance.

## Supplemental Information

Document S1. Figures S1–S7

Document S2. Article plus Supplemental Information
