# Supplementary material for: Myosin VI-Dependent Actin Cages Encapsulate Parkin-Positive Damaged Mitochondria
Source: Dev Cell. 2018 Feb 26;44(4):484–499.e6. doi: 10.1016/j.devcel.2018.01.007 (PMC5932465; doi:10.1016/j.devcel.2018.01.007)
Supplement: Document S1. Figures S1–S7 [file mmc1.pdf]

Developmental Cell, Volume *44*

Supplemental Information

Myosin VI-Dependent Actin Cages Encapsulate Parkin-Positive Damaged Mitochondria

Antonina J. Kruppa, Chieko Kishi-Itakura, Thomas A. Masters, Joanna E. Rorbach, Guinevere L. Grice, John Kendrick-Jones, James A. Nathan, Michal Minczuk, and Folma Buss

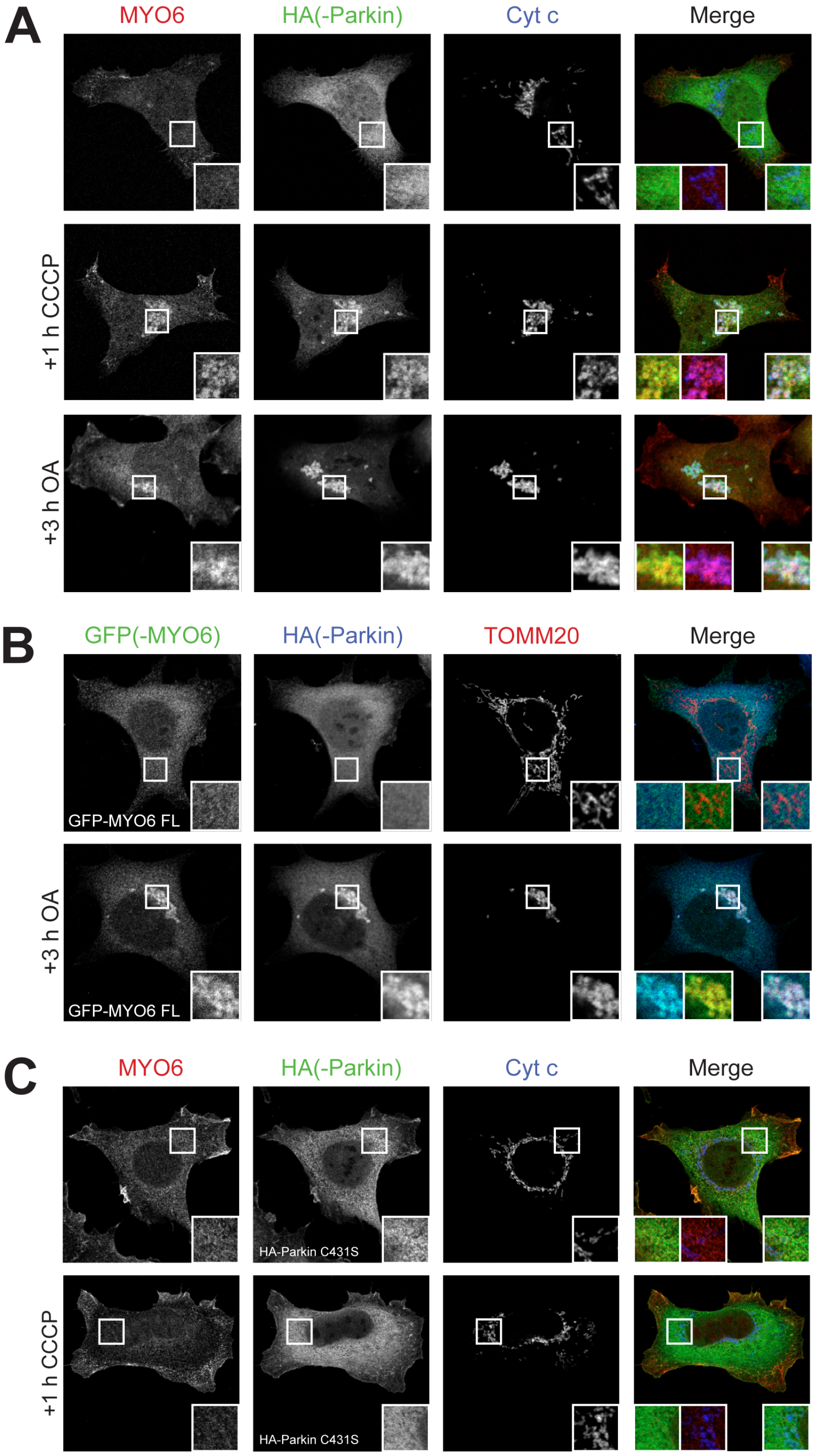

Figure S1, Related to Figure 1. Endogenous and GFP-tagged MYO6 are recruited to damaged mitochondria after oligomycin/antimycin A treatment only in the presence of wild-type Parkin.
(A) HEK293 cells stably expressing HA-Parkin were incubated for 1 h with 10 μM CCCP, for 3 h with a combination of 10 μM oligomycin and 4 μM antimycin A (OA), or left untreated. Images were acquired by confocal microscopy after staining for endogenous MYO6, HA to detect Parkin, and cytochrome c (Cyt c) to visualize mitochondria. (B) HEK293 cells stably expressing HA-Parkin transiently transfected with full-length (FL) GFP-MYO6 were left untreated or incubated for 3 h with OA. Images were acquired by confocal microscopy after staining for the GFP tag on MYO6, HA to detect Parkin, and TOMM20 to label mitochondria. (C) HEK293 cells stably expressing catalytically inactive HA-Parkin harboring the C431S mutation were left untreated or incubated for 1 h with 10 μM CCCP. Cells were processed for immunofluorescence as in (A) and imaged by confocal microscopy. Images in (A–C) are representative of three independent experiments.

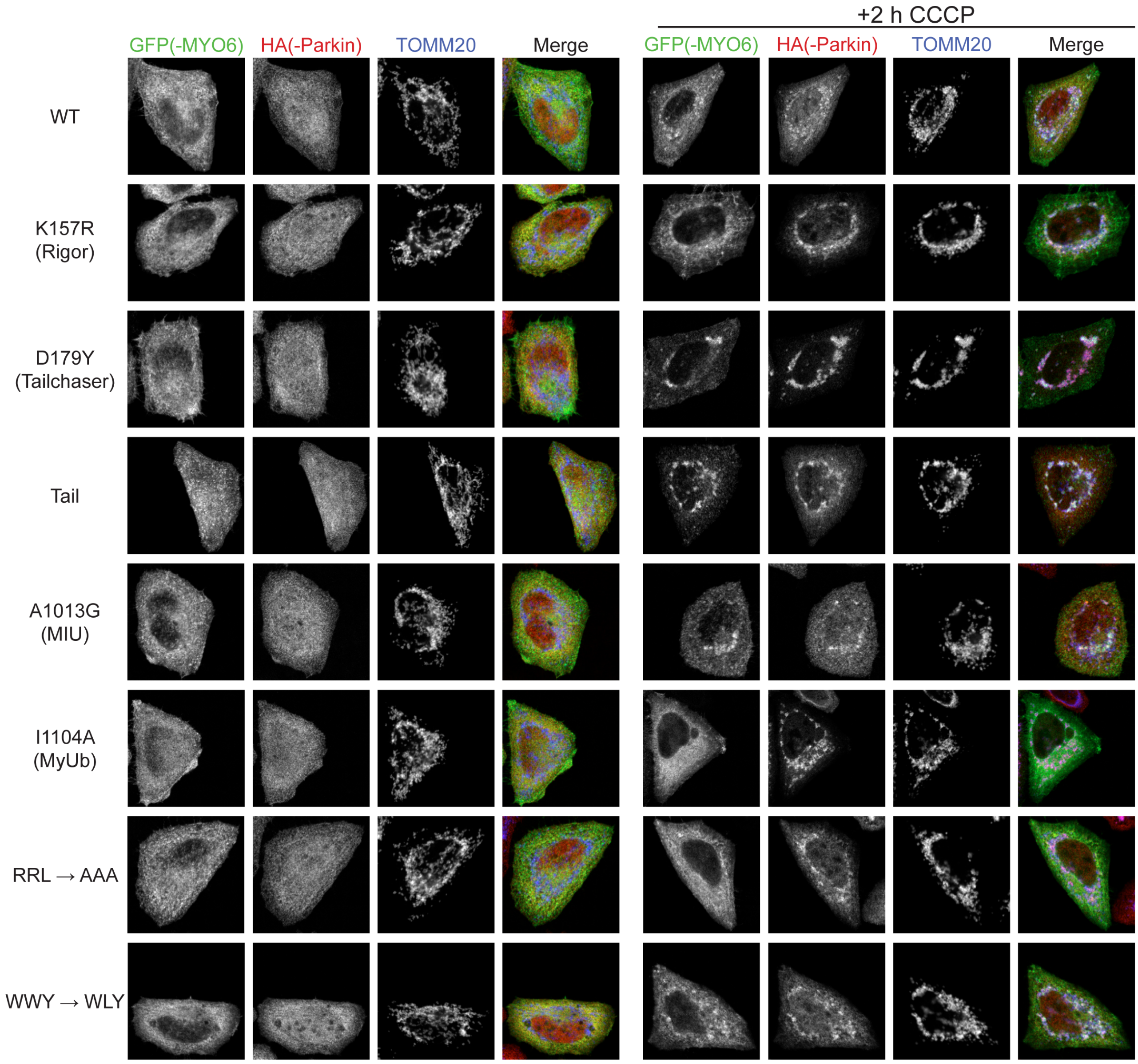

Figure S2, Related to Figure 2. Representative images of GFP-tagged wild-type or mutant MYO6 recruitment to damaged mitochondria.
HeLaM cells stably expressing HA-Parkin transiently transfected with wild-type (WT) or the indicated mutant versions of the GFP-MYO6 construct were left untreated or incubated for 2 h with 10 μM CCCP. Representative images of cells acquired by confocal microscopy after staining for the GFP tag on MYO6, HA to detect Parkin, and TOMM20 to label mitochondria are shown from more than three independent experiments.

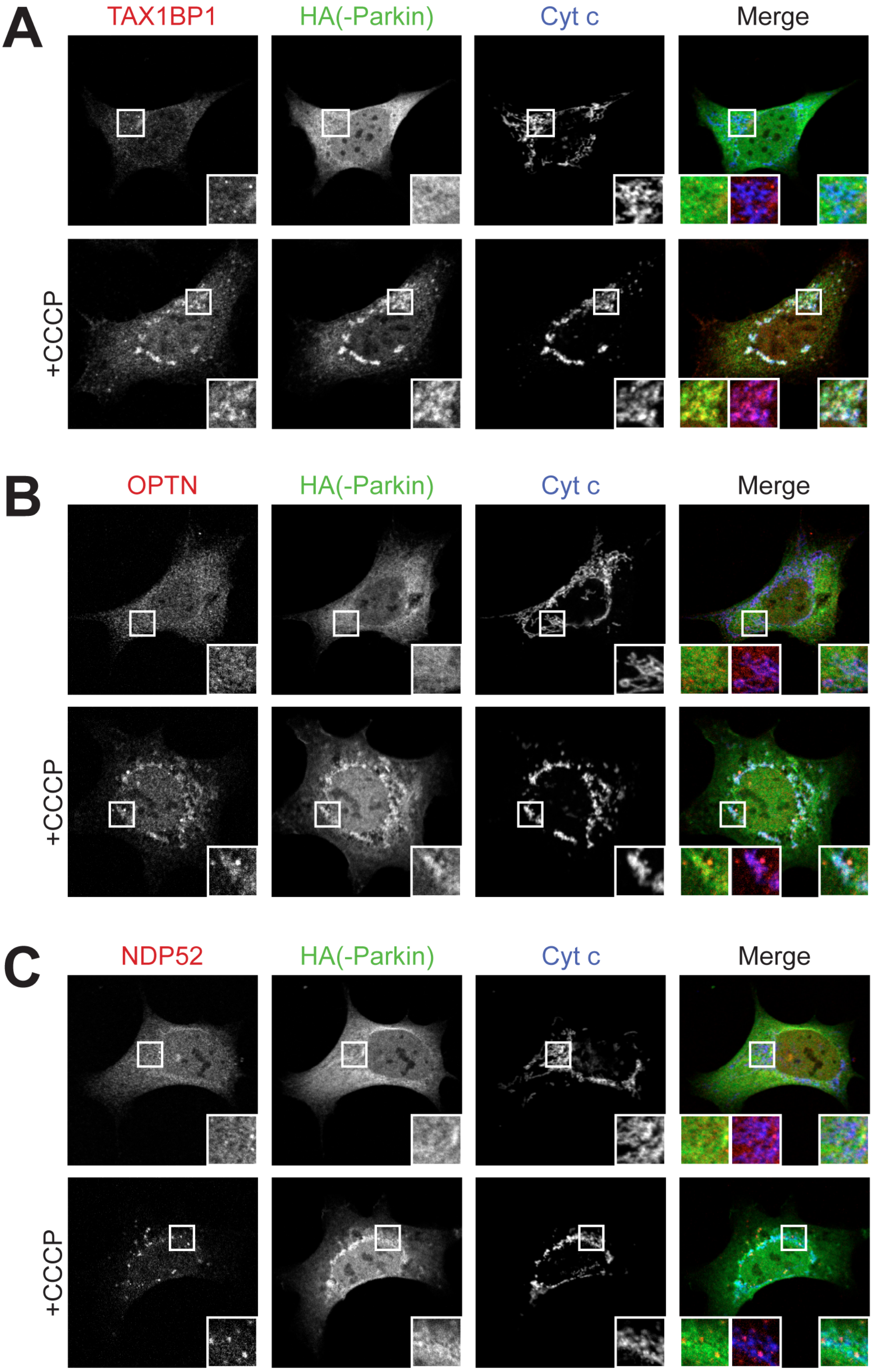

Figure S3, Related to Figure 2. Recruitment of endogenous autophagy receptors to damaged mitochondria.
HEK293 cells stably expressing HA-Parkin were left untreated or incubated for 1 h with 10 μM CCCP. Images were acquired by confocal microscopy after staining for endogenous TAX1BP1 (A), OPTN (B) or NDP52 (C), HA to detect Parkin, and cytochrome c (Cyt c) to visualize mitochondria. Images are representative of three independent experiments.

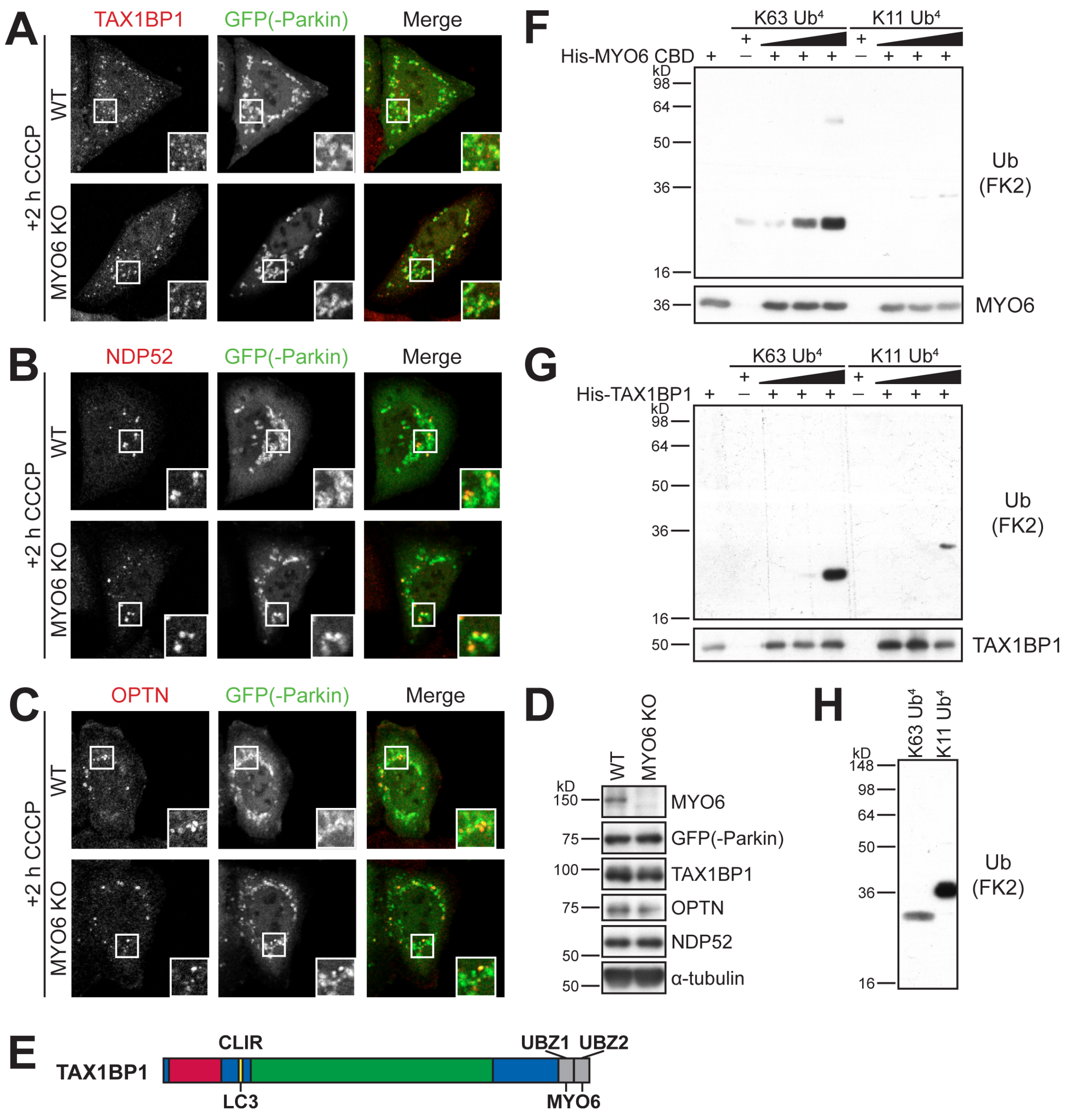

Figure S4, Related to Figure 2. The autophagy receptors are recruited independently from MYO6 and *in vitro* binding assay of MYO6 and TAX1BP1 to ubiquitin chains.
(A–D) Wild-type (WT) or MYO6 knockout (KO) HeLaM cells were transiently transfected with GFP-Parkin and incubated for 2 h with 10 μM CCCP. Images were acquired by confocal microscopy after staining for endogenous TAX1BP1 (A), NDP52 (B) or OPTN (C) and the GFP tag on Parkin. (D) Immunoblotting of lysates corresponding to (A–C) confirming complete loss of MYO6, overexpression of GFP-Parkin, and similar levels of autophagy receptors. α-tubulin is shown as a loading control. (E) Illustration of TAX1BP1 domain organization: SKICH domain (red), non-canonical LIR (CLIR, yellow), coiled-coil region (green), and two ubiquitin-binding zinc fingers (UBZ, grey). (F) His-tagged MYO6 cargo binding domain (CBD) or (G) His-tagged TAX1BP1 (C-terminal half) were bound to Ni-NTA agarose and incubated with increasing concentrations of K63 or K11 tetra-ubiquitin (Ub^4). Bound proteins were visualized by immunoblotting using antibodies against ubiquitin (FK2) and MYO6 or TAX1BP1, respectively. (H) Equal amounts of K63 and K11 Ub^4 chains were immunoblotted using an antibody against ubiquitin (FK2). Images in (A–C, F, G) are representative of three independent experiments.

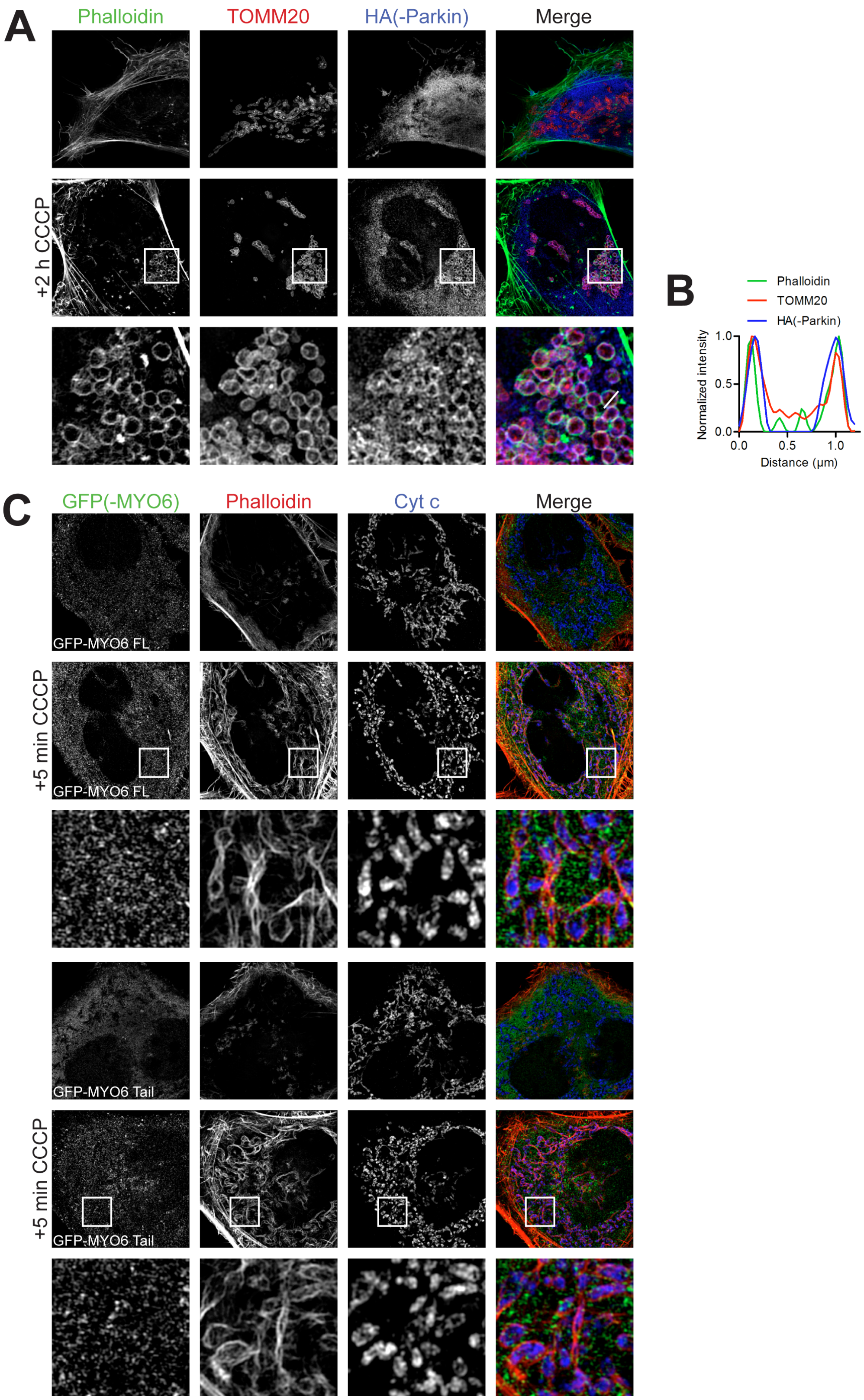

Figure S5, Related to Figure 3. Different F-actin structures on mitochondria after 5 min or 2 h of CCCP treatment.

(A) HEK293 cells stably expressing HA-Parkin were left untreated or incubated for 2 h with 10 µM CCCP. Images were acquired by SR-SIM after staining F-actin with phalloidin, TOMM20 to visualize the outer mitochondrial membrane, and HA to detect Parkin. (B) Line profile of Parkin-positive mitochondrion that is actin-positive along white line indicated in (A). (C) HeLaM cells stably expressing HA-Parkin transiently transfected with GFP-MYO6, either full-length (FL) or tail, were left untreated or incubated for 5 min with 10 µM CCCP. Images were acquired by SR-SIM after staining for the GFP tag on MYO6, with phalloidin to visualize F-actin, and cytochrome c (Cyt c) as a mitochondrial marker. Images in (A, C) are representative of three independent experiments.

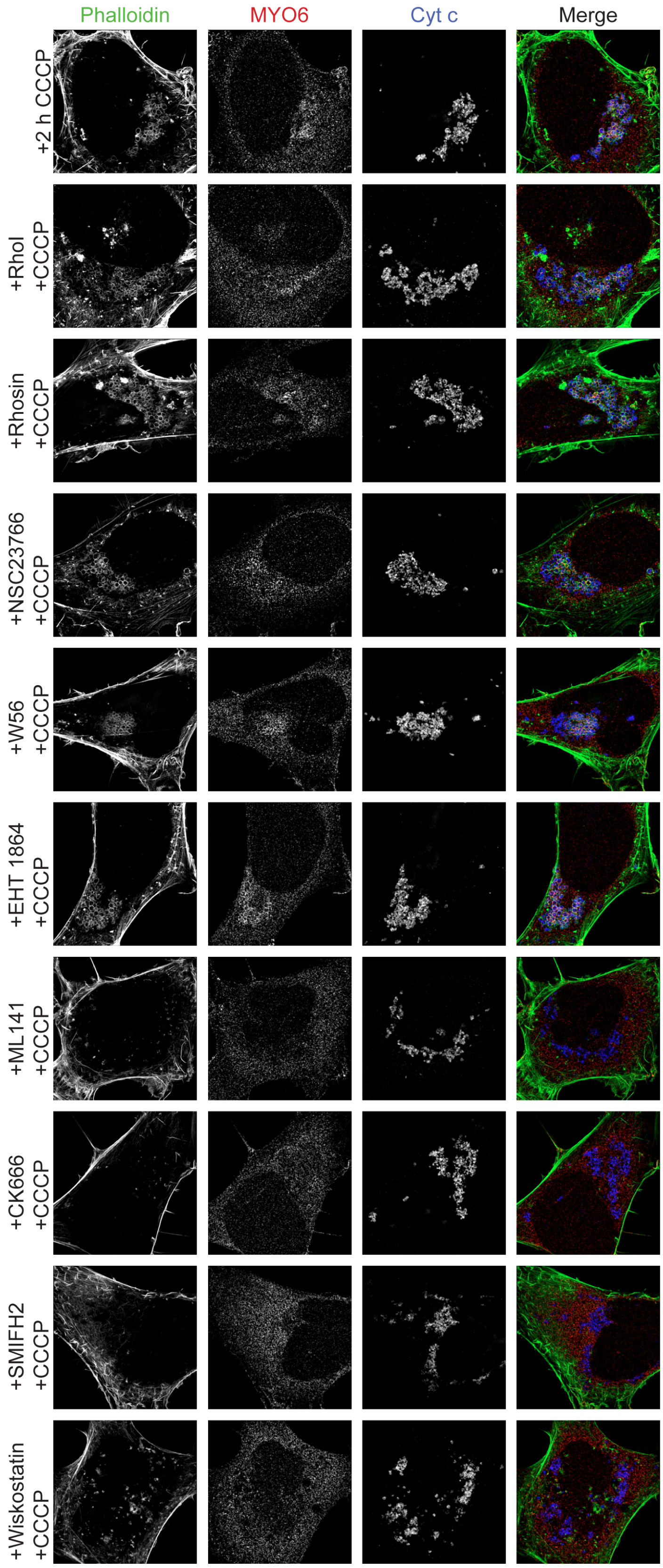

Figure S6, Related to Figure 4. The effect of different actin polymerisation inhibitors on actin cage formation around damaged mitochondria.

HEK293 cells stably expressing HA-Parkin were incubated for 2 h with 10 μM CCCP and inhibitors of actin regulators Rho (0.5 μg/ml Rho inhibitor I, RhoI, or 50 μM Rhosin), Rac1 (100 μM NSC23766, 100 μM W56, or 10 μM EHT 1864), or cdc42 (20 μM ML141), and actin nucleators Arp2/3 complex (100 μM CK666), formins (20 μM SMIFH2), or N-WASP (5 μM Wiskostatin). Images were acquired by SR-SIM after staining with phalloidin to visualize F-actin, endogenous MYO6, and with cytochrome c (Cyt c) to label mitochondria and are representative of more than three independent experiments.

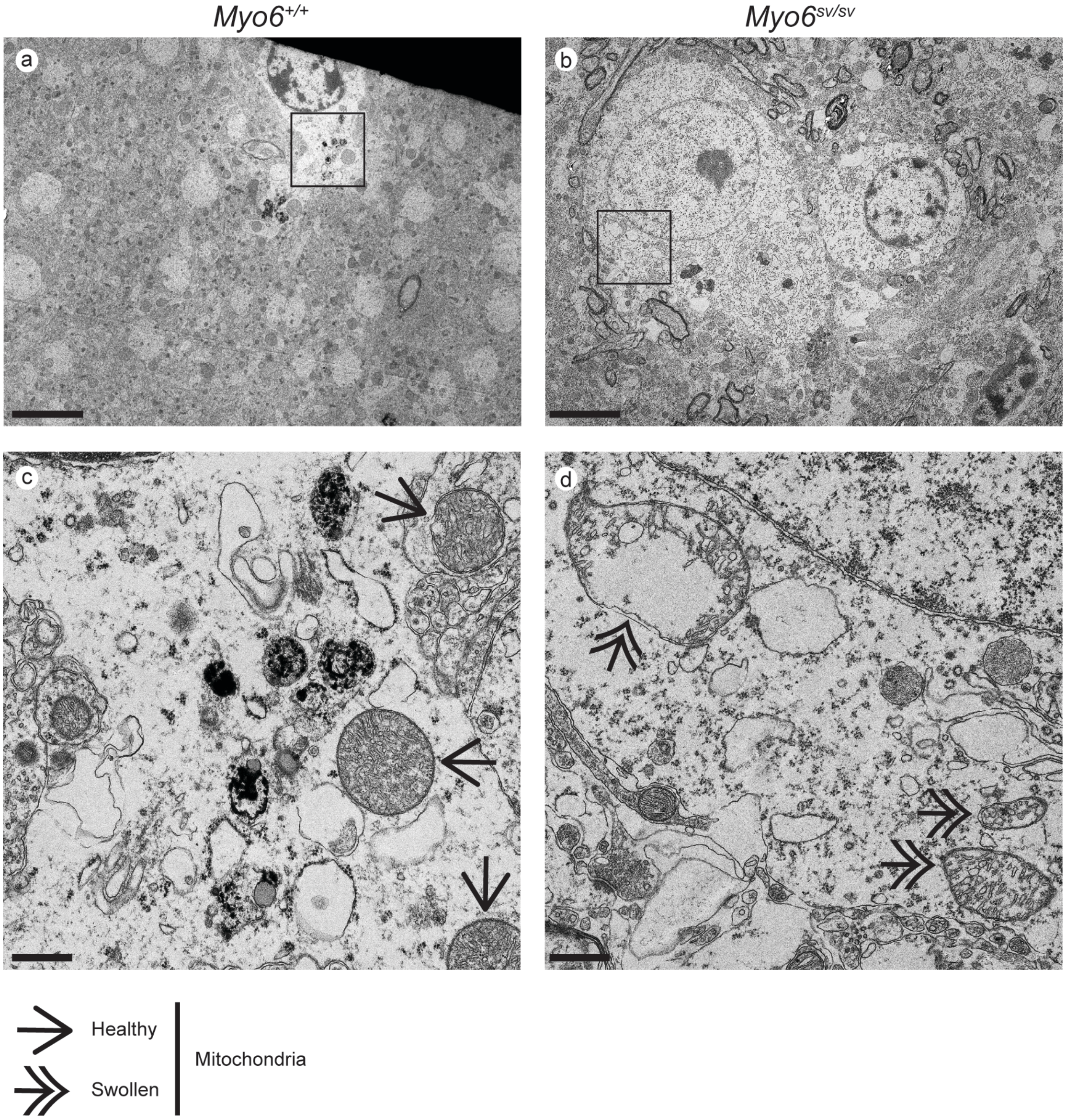

Figure S7, Related to Figure 6. Mitochondria appear swollen in *Snell's waltzer* astrocytes from the hippocampal cortex.

Sixteen-month old wild-type (*Myo6*$^{+/+}$) and *Snell's waltzer* (*Myo6*$^{sv/sv}$) mice were starved overnight and the brains processed for conventional electron microscopy. The single-headed arrows point to healthy mitochondria, while the double-headed arrows indicate swollen mitochondria. Scale bars: 5 μm (panels a and b), 500 nm (panels c and d).
